# Corporate digital transformation, internal control and total factor productivity

**DOI:** 10.1371/journal.pone.0298633

**Published:** 2024-03-20

**Authors:** Xiao Li, Feiyang Zhao, Zhiquan Zhao

**Affiliations:** 1 Systems and Industrial Engineering Technology Research Center, Zhongyuan University of Technology, Zhengzhou, Henan, China; 2 UCD Michael Smurfit Graduate Business School, University College Dublin, Dublin, Ireland; 3 School of Economics and Management, Zhongyuan University of Technology, Zhengzhou, Henan, China; Krirk University, THAILAND

## Abstract

Based on Resource-based theory and Internal Control (IC) theory, this study elucidates the impacts of corporate digital transformation on total factor productivity, and IC effectiveness, as well as the mechanism among digital transformation, IC and total factor productivity. The results show that digital transformation promotes total factor productivity and IC effectiveness. And effective IC has a significant mediating effect for the impact of digital transformation on total factor productivity. Heterogeneity discussion shows that compared with high-tech enterprises, in non-high-tech ones, digital transformation increases total factor productivity, and more significantly enhances IC effectiveness, presenting a mechanism that digital transformation facilitates IC, and increases total factor productivity. For non-high-tech enterprises, with higher heterogeneity of executive education backgrounds, digital transformation promotes IC effectiveness and total factor productivity, showing the transmission effect among digital transformation, IC and total factor productivity. Finally, it is suggested that the regulatory authorities advance digital infrastructure construction, to reinforce IC and risk prevention, thereby increase total factor productivity. And enterprises grasp the opportunity of digital economy development, promote the mechanism that digital transformation facilitates IC effectiveness, and increases total factor productivity. Non-high-tech ones motivate digital elements’ governance efficacy, optimize executive structure, coordinately promote digital strategy, and help the national economy acquire high-quality development. The study provides enlightenments to achieve high-quality development.

## 1. Introduction

With artificial intelligence, big data, cloud computing, and other digital technologies, digital transformation has become the core of corporate survival and development. More and more enterprises are developing big data, mobile internet and artificial intelligence, to upgrade industrial digitization. Digital technologies may lead to strategic risks, and increase uncertainty [1, 2]. In contrast, Rothberg and Erickson (2017) [3], Cenamor et al. (2019) [4] believed that big data and artificial intelligence changed corporate traditional business mode and improved operational efficiency. In fact, digital transformation means business structure construction, workflow overall optimization and fundamental changes, through information technologies and innovation [5], building a value creation system with data as core driving factor, and realizing business mode transformation [6]. As an emerging economy, China’s economy has entered a new normal, shifting from high-speed growth to high-quality development. Improving total factor productivity is a key to acquire high-quality economic development [7]. The total factor productivity is the additional production efficiency under given factor inputs [8], measuring the quality and sustainability of macroeconomic growth, as well as micro-enterprises’ operating efficiency and development quality [9]. The improvement of total factor productivity is an important symbol for corporate transformation and upgrading. With gradual integration of digital and real economies, it is of great significance to explore the impact of digital transformation on total factor productivity for promoting high-quality economic development.

Based on Resource-based theory, valuable and scarce resources are crucial to acquire competitive advantages [10]. Digital capability is an important intangible resource that is difficult to imitate, and cannot be replaced [11]. Then, at this stage, how does digital transformation affect total factor productivity? Corporate digital transformation is a long-term and gradual process [12, 13]. Goldfarb and Tucker (2019) [14] believed that digital technologies promotes IC processes more transparent. However, Wu et al. (2014) [15] argued that superior information technology capabilities lack significant effects on asset security and business legal compliance. In 2008, Chinese Ministry of Finance and other four ministries and commissions issued the “Basic Norms for Corporate Internal Control”, emphasizing that enterprises should adopt information technologies to ameliorate IC construction, build information systems that are suitable for business activities, organically integrate IC processes and information systems, promote automatic business controls, and eliminate improper manipulation. With the rapid development of digital economy, the transformation from informatization to digitization is an inevitable trend. And IC is the measures, methods and procedures that connect and restrict business activities. Then, can digital transformation enhance IC effectiveness? The “Basic Norms for Corporate Internal Control” clearly points out that IC reasonably ensures business legality and compliance, assets safety, financial reports’ truthfulness and integrity, improves operational efficiency and effect, and promotes enterprises to realize development strategies. And total factor productivity reveals the factors that are difficult to observe such as management skills, institutional innovation and growth potentials [9], and it is an important indicator to evaluate corporate development quality [16]. So, at present, how does corporate IC affect total factor productivity? Further, is there an intrinsic mechanism among digital transformation, IC and total factor productivity?

The economic prosperity and recession affect the trend of the stock markets, which are the “barometers” of the national economy. In accordance with the “Company Act”, the IPO of an incorporated company must be approved by the State Council or the securities regulatory department authorized by the State Council. Compared with non-listed companies, listed companies have more stringent requirements for financial disclosure. Based on the above practical considerations, given data availability, this study takes the listed enterprises in Shanghai and Shenzhen stock markets from 2012 to 2021 as the sample, in line with Resource-based theory and IC theory, explains the impacts of digital transformation on total factor productivity, and IC effectiveness, as well as the internal mechanism among digital transformation, IC, and total factor productivity, to provide empirical evidence for advancing digital transformation, facilitating IC and total factor productivity. The remaining parts are organized as follows. Section **2** provides Literature review, theoretical analysis, and research hypothesis. Section **3** presents Data source, variable definition and model setting. Section **4** shows Descriptive statistic and correlation. Section **5** carries out Model regression analysis. Section **6** conducts Robustness test. Section **7** displays Heterogeneity discussion. And Section **8** draws conclusions and recommendations.

The possible contributions are as follows. Firstly, existing studies explored the impacts of digital technologies on IC effectiveness [14, 17], operational process management [4, 18], and total factor productivity [19]; and that of IC on operational efficiency [20]. However, few literatures delve into the mechanism among digital transformation, IC, and total factor productivity. Based on the effective combination of Resource-based theory and IC theory, this study explains the mediating effect of IC between digital transformation and total factor productivity, contributing to clarify the intrinsic mechanism that digital transformation enables total factor productivity. Secondly, with digital transformation, enterprises utilize information, computing, communication and connection technologies to improve operation [5], presenting heterogeneous effects on total factor productivity [21]. However, few literatures explore the digitalization effects from corporate technical attributes. This study distinguishes high-tech from non-high-tech enterprises, and examines the heterogeneous effects of digital transformation on IC effectiveness, and total factor productivity, as well as the heterogeneous mechanism among them, enriching relevant literatures on digital transformation enablement. Thirdly, digitalization has transformed fuzzy decisions into scientific decisions assisted by big data [22], and executives’ diverse education backgrounds enables them to rationally analyze problems from different perspectives [23]. In light of these, for non-high-tech enterprises, this study discriminates the heterogeneity of executive education backgrounds, elucidates the heterogeneous effects of digital transformation on IC and total factor productivity respectively, and the heterogeneous mechanism among them, providing practical enlightenments for advancing high-quality development in emerging markets.

## 2. Literature review, theoretical analysis and research hypothesis

### 2.1 Digital transformation and total factor productivity

With the application of digital technologies, the face-to-face coordination based on on-site management is gradually replaced with digital virtual space [14]. With regard to digital transformation and total factor productivity, existing studies have not reached a consensus. Some scholars believe that new digital technologies may bring strategic risks [2], increase uncertainty in identifying, measuring, recording and reporting transactions and events [1]. Although digital transformation expands access to resources, along with information channels’ continuous expansion, the exponential increase in network connections and information deluge are emerging, leading to difficulties to discover and capture valuable contents timely from massive information [24], resulting in information overload, and the mismatch between information and corporate management, thus inducing “ability curse” and raising decision-making threshold. Instead, from the “data-driven” perspective, digital technologies simplify administrative procedures, facilitate real-time communication between employees, between enterprises and suppliers, as well as consumers, and partners, then improve information acquisition efficiency [25], promote internal specialization, enhance production efficiency, and ultimately increase total factor productivity [19].

In the context of digital transformation, enterprises should not only have the ability to quickly collect real-time data, but also analyze data in time, to translate data analyses into effective risk assessment, strategy making and business decisions [26]. Based on Resource-based theory, big data and artificial intelligence are changing traditional business modes [3], enhancing corporate market positioning, and improving operational efficiency through optimized process management or advanced market knowledge [4]. Ameliorating resource allocation and information integration, digital transformation advances traditional growth drivers, and cultivates new driving forces [19]. By embedding digital technologies in production and service, enterprises optimize original business processes, improve organizational structure, and reduce operational costs [27]. Digital transformation breaks internal departments’ boundaries, realizes real-time and transparent whole-process monitoring in organizational operation, improves information transfer and processing efficiency, and implements efficient operation [18]. With digital transformation, digital management system is embedded in each link in operation [28], improving business processes, and achieving refined production [29]. Further, automated production costs are reduced [30], intelligent and flexible operation processes are realized, production cycle is shortened, defective index is decreased, and capital operation efficiency is enhanced [5]. And the network supply chain superior to integrated configuration is formed [31], so as to increase total factor productivity.

Based on the above analyses, the following research hypothesis is proposed.

**Hypothesis 1.** Digital transformation significantly increases total factor productivity.

### 2.2 Digital transformation, IC effectiveness, and total factor productivity

With digital industrialization and industrial digitization, digital transformation is an inevitable choice for enterprises to adapt to economic and social development. Digital transformation means innovating production modes, and reshaping production structure [32], deeply integrating digital technologies with operational activities, and systematically changing business processes, products, services and business ecology [33], which may result in that IC is difficult to adapt to in-depth changes immediately, unable to comprehensively collect information and effectively assess risks, thus leading to IC failure. For instance, outstanding IT capability has no significant effects on assets safety, and business legal compliance [15]. In contrast, Goldfarb and Tucker (2019) [14] believed that digital technologies significantly enhanced internal information transparency, reduced improper operation manipulation, improved stakeholders’ ability to supervise information, and then enhanced IC effectiveness. From internal governance perspective, digital technologies are embedded into corporate contract arrangement, give play to risk control advantages, enhance IC digitalization, and fortify IC execution efficiency, thus facilitating IC effectiveness [17].

The management ideas and IC methods endowed by digital technologies are gradually embedded in daily operation, facilitating IC processes more transparent [14]. Based on Resource-based theory, digital technologies are embedded into business processes, enabling management decisions’ effective communication and timely feedback. Enterprises adopt big data technology to analyze and monitor operation, restrain irrational behaviors driven by self-interest motive, alleviating agency problems. With digital technology support, the real-time monitoring on key links in operation is conducted through embedded automatic system, shifting from traditional post-verification to pre-prevention and in-process controls. Digital transformation creates an internal environment for collaborative control, improves the efficiency of risk assessment and response, promotes control activities’ intellectualization, and improves the timeliness and effectiveness of information communication. Digital transformation bridges internal and external information communication, effectively alleviating information asymmetry and delay [34], improving information environment, and enhancing minority shareholders’ willingness and ability to participate in corporate governance [35], and motivating enterprises to actively build accounting and management information, decision support and artificial intelligence systems, to make information more fair and reliable, control more automated, and decision support more valuable. Moreover, digital transformation presents characteristics such as being not easy to tamper with, authority checks and balances, process embedding, man-machine collaboration, etc., realizing more effective information processing, mitigating governance risks caused by information asymmetry [36], avoiding managers from overriding effective IC, and reducing control risks, to ensure that IC effectiveness is enhanced on the whole.

Based on the above analyses, the following research hypothesis is proposed.

**Hypothesis 2.** Digital transformation enhances IC effectiveness significantly.

Digital technologies realize real-time and transparent operation and management [37], and fully enhance IC effectiveness. As an important governance mechanism, IC arises from management needs. Its basic functions involve check and constraint, protection and guidance, supervision and influence, measurement and evaluation [38]. Effective IC affords an important guarantee for achieving high-quality development. Based on IC theory, IC runs through decision making, implementation and supervision, including five elements, namely, internal environment, risk assessment, control activity, information and communication, and internal supervision [39]. Internal environment is a basis for IC execution, defining decision making, execution, supervision and other responsibilities, and forming a scientific responsibilities division mechanism. Through checks and balances, the good internal environment achieves mutual constraints and supervision in institutions setting, rights and responsibilities allocation, business processes, reducing agency costs, thereby improves organizational management efficiency. And risk assessment is vital for identifying, analyzing, and dealing with business risks. Effective risk assessment promptly identifies the risks related to human resource, management, financial factors, etc., and combines preventive with discovery control activities, to control risks within an acceptable range, ensure risk response sustainability, and improve resources’ optimal allocation [40]. Adequate information and communication are beneficial for risk response, alleviating information asymmetry, reducing information communication costs, and advancing contract execution efficiency. Good internal supervision prevents managers from avoiding risky investments, weakens opportunistic behaviors to a greater extent, and optimizes internal management. Therefore, effective IC alleviates agency conflicts [41], improves production efficiency [20], and increases total factor productivity. Further, considering Hypothesis 2 that digital transformation enhances IC effectiveness, this study argues that digital transformation reduces potential control risks, optimizes organization structure, upgrades business pattern, ameliorates resource allocation, and hoists core competitiveness, forming a transmission pathway that digital transformation enhances IC effectiveness, and increases total factor productivity.

Based on the above analyses, the following research hypothesis is proposed.

**Hypothesis 3.** Effective IC has a significant mediating effect for the impact of digital transformation on total factor productivity.

## 3. Data source, variable definition, and model setting

### 3.1 Data source

In 2012, China Securities Regulatory Commission issued the revised “Guidance on Listed Companies’ Industry Classification”. To facilitate industry classification, the listed enterprises publicly traded in Shanghai and Shenzhen stock markets from 2012 to 2021 are taken as the sample. The data are from Wind financial terminal, and “DIB · IC Index of Listed Companies” database. To ensure integrity and reliability, the observations with missing data are eliminated. Given the particularity of financial statements in the financial industry [42], the observations in this industry are excluded. And the observations processed by ST, and *ST are eliminated. Finally, the disclosed data of 2773 enterprises are obtained as effective observations. In addition, the bidirectional 1% quantiles Wionsorize processing are conducted for continuous variables, to reduce outliers’ adverse influence (S1 Dataset).

### 3.2 Variable definition

Table 1 presents the variables’ names and descriptions.

**Table 1:** Variable name and description.

Nature	Symbol	Name	Calculation method
Explained variable	TFP_Fe	Total factor productivity	Acquired from Eqs (1) and (2)
Explanatory variable	DIGIT	Corporate digital transformation	Obtained from text analyses
Mediating variable	IC	IC effectiveness	DIB · IC Index
Control variable	LEV	Asset-liability ratio	Total liabilities/total assets
	TURNOVER	Total assets turnover	Current operating revenue/average total assets at the beginning and end of this period
	ROA	Return on assets	Net income/average total assets at the beginning and end of this period
	TQ	Corporate growth	Corporate market value/book value
	INDED	Board structure	The proportion of independent directors on the board
	R&D	R&D investment	The percentage of R&D input to operating income
	Concen	Ownership concentration	The shareholding ratio of the largest shareholder/that of the second largest shareholder
	Age	Company age	The periods from corporate establishment to the end of the observation year
	LnSALARY	Executive compensation	The natural logarithm of top three executive compensations
	LnASSET	Corporate scale	The natural logarithm of total assets at the end
	Big4	“Big four” audit	Dummy variable, 1 for the international “Big four” accounting firms; otherwise, 0
	SOE	Property attribute	Dummy variable, 1 for state-owned enterprises; otherwise, 0
	YEAR	Year	Annual effect
	IND	Industry	Industry effect; dummy variables set in accordance with the “Guidance on Listed Companies’ Industry Classification”
	ε		Random disturbance term

#### 3.2.1 Explained variable

Total factor productivity is an important indicator reflecting corporate value [43]. It evaluates the contributions of system optimization, management level, technology introduction, etc., other than factor input quantity. With reference to Lu and Lian (2012) [44], the following fixed-effects Model 1 is adopted to measure total factor productivity (TFP_Fe), approximately.

Model 1.


$$LnY_{i,t}=\zeta_{0}+\zeta_{1}LnK_{i,t}+\zeta_{2}LnL_{i,t}+\zeta_{3}LnM_{i,t}+\zeta_{4}\sum_{t}REGION+\zeta_{5}\sum_{t}YEAR+\zeta_{6}\sum_{t}IND+\varepsilon_{i,t}$$ (1)

In Eq (1), for company ***i*** in year ***t***, Y_i,t_, K_i,t_, L_i,t_, and M_i,t_ represent the total output measured with operating revenue, capital input measured with net fixed assets, labor input, and intermediate input, respectively. REGION, YEAR, IND indicate the region, year, and industry dummy variables. The regression is conducted for Model 1, to obtain the estimated coefficients on LnK_i,t_, and LnL_i,t_. And the coefficients are substituted into Eq (2) to calculate TFP_Fe.


$$TFP\_Fe_{i,t}=LnY_{i,t}-{\hat{\zeta }}_{1}LnK_{i,t}-{\hat{\zeta }}_{2}LnL_{i,t}$$ (2)

#### 3.2.2 Explanatory variable

As a major strategy for high-quality development, digital transformation is more likely to be reflected in annual reports with summary and guidance [45]. With reference to Yuan et al. (2021) [19], Xiao et al. (2022) [46], and Li et al. (2023) [47], text analyses are adopted to obtain the degree of digital transformation (DIGIT). Firstly, based on the semantic expression of national policies related to digital economy, the websites of the State Council, and the Ministry of Industry and Information Technology are searched, to obtain 32 important national digital economy-related policy documents released during 2012–2021, and extract keywords related to digitalization. After Python words segmentation and manual recognition, a total of 197 words with more than 4 frequencies are selected, such as “artificial intelligence, digitalization, intelligence, and key technology.” S1 Appendix lists these keywords, which are adopted to construct a relatively objective and complete digital glossary. Secondly, the 197 words in the digital glossary are extended to the “Jieba” Chinese lexicon in Python software package. Thirdly, given that listed companies’ annual reports are open and reliable, and there is no obvious motivation for text manipulation in “Management Discussion and Analysis (MD&A)” [48], text analyses are utilized to segment MD&A content, to obtain the frequencies of 197 digital keywords. Finally, in view of the differences in MD&A text length, the frequencies are divided by MD&A length, to measure DIGIT. The bigger the DIGIT, the higher the degree of digital transformation.

#### 3.2.3 Mediating variable

With reference to Li (2022) [38], Li and Zhao (2022) [39], the “DIB · IC Index of Listed Companies” is adopted to measure IC effectiveness. This index is released by Shenzhen Dibo Enterprise Risk Management Technology Co., Ltd., and reflects IC levels and risk prevention capabilities, comprehensively manifests control objectives’ realization and IC effectiveness. The higher the index, the more effective the IC.

#### 3.2.4 Control variable

In line with Goldfarb and Tucker (2019) [14], Yuan et al. (2021) [19], and Li (2022) [38], Asset-liability ratio, Total assets turnover, Return on assets, Corporate growth, Board structure, R&D investment, Ownership concentration, Company age, Executive compensation, Corporate scale, “Big four” audit, and Property attribute are taken as the control variables, to examine the possible impacts on total factor productivity, and IC effectiveness. Also, annual and industry effects are controlled in regression.

### 3.3 Model setting-up

With reference to Goldfarb and Tucker (2019) [14], Yuan et al. (2021) [19], and Li (2022) [38], the following Models 2 to 4 are constructed, to examine Hypotheses 1 to 3 mentioned above. Digital transformation is a gradual process [12, 13], which is not achieved overnight. In Models 2 to 4, for the explanatory variable, the current value is first adopted for regression, and in Robustness test, the first-order lag is considered for analyses. In the control variables, LEV, TURNOVER, ROA, TQ, R&D, LnSALARY, LnASSET and Big4 are taken as first-order lags, to mitigate the endogeneity caused by reverse causality. Moreover, to avoid adverse effects caused by excessive dimensional differences between the explained and explanatory variables, from Models 2 to 4, DIGIT equals to the degree of digital transformation from text analyses multiplied by 10. And in Model 3 and 4, IC is the value of “DIB · IC Index” divided by 1000.

Model 2.


$$\begin{array}{l} TFP\_Fe_{i,t}=\alpha_{0}+\alpha_{1}DIGIT_{i,t}+\alpha_{2}LEV_{i,t-1}+\alpha_{3}TURNOVER_{i,t-1}+\alpha_{4}ROA_{i,t-1}+\alpha_{5}TQ_{i,t-1}+\\ \;\alpha_{6}INDED_{i,t}+\alpha_{7}R\&D_{i,t-1}+\alpha_{8}Concen_{i,t}+\alpha_{9}Age_{i,t}+\alpha_{10}LnSALARY_{i,t-1}+\\ \;\alpha_{11}LnASSET_{i,t-1}+\alpha_{12}Big4_{i,t-1}+\alpha_{13}SOE_{i,t}+\\ \;\alpha_{14}\sum_{t}YEAR+\alpha_{15}\sum_{t}IND+\varepsilon_{i,t} \end{array}$$ (3)

Model 3.


$$\begin{array}{l} IC_{i,t}=\beta_{0}+\beta_{1}DIGIT_{i,t}+\beta_{2}LEV_{i,t-1}+\beta_{3}TURNOVER_{i,t-1}+\beta_{4}ROA_{i,t-1}+\beta_{5}TQ_{i,t-1}+\\ \;\beta_{6}INDED_{i,t}+\beta_{7}R\&D_{i,t-1}+\beta_{8}Concen_{i,t}+\beta_{9}Age_{i,t}+\beta_{10}LnSALARY_{i,t-1}+\\ \;\beta_{11}LnASSET_{i,t-1}+\beta_{12}Big4_{i,t-1}+\beta_{13}SOE_{i,t}+\\ \;\beta_{14}\sum_{t}YEAR+\beta_{15}\sum_{t}IND+\varepsilon_{i,t} \end{array}$$ (4)

Model 4.


$$\begin{array}{l} TFP\_Fe_{i,t}=\delta_{0}+\delta_{1}DIGIT_{i,t}+\delta_{2}IC_{i,t}+\delta_{3}LEV_{i,t-1}+\delta_{4}TURNOVER_{i,t-1}+\delta_{5}ROA_{i,t-1}+\\ \;\delta_{6}TQ_{i,t-1}+\delta_{7}INDED_{i,t}+\delta_{8}R\&D_{i,t-1}+\delta_{9}Concen_{i,t}+\delta_{10}Age_{i,t}+\\ \;\delta_{11}LnSALARY_{i,t-1}+\delta_{12}LnASSET_{i,t-1}+\delta_{13}Big4_{i,t-1}+\\ \;\delta_{14}SOE_{i,t}+\delta_{15}\sum_{t}YEAR+\delta_{16}\sum_{t}IND+\varepsilon_{i,t} \end{array}$$ (5)

**Fig 1:**
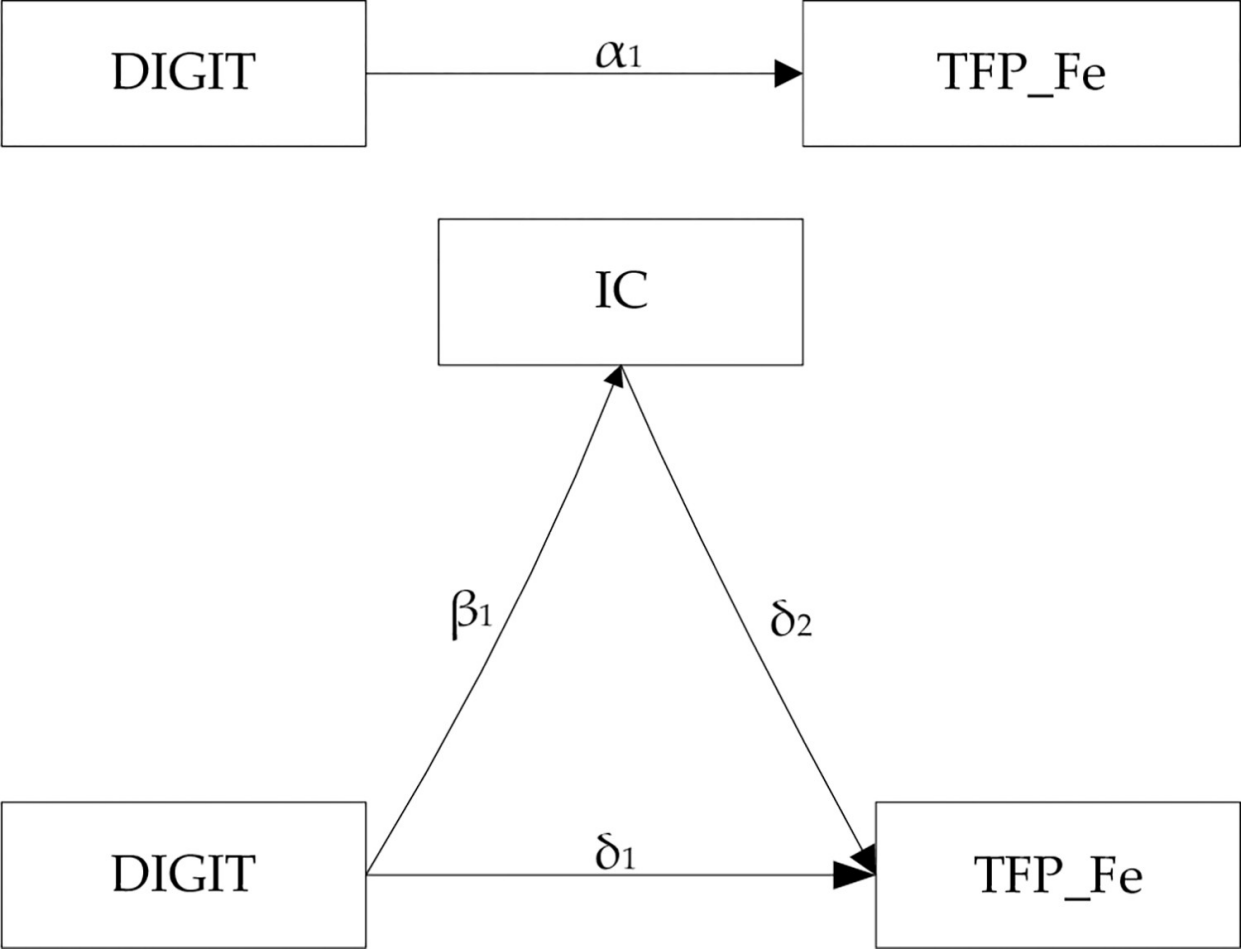
Mediating effect diagram.

In Model 2, the coefficient α_1_ represents the total effect of digital transformation on total factor productivity. In Model 3, β_1_ is the effect of digital transformation on IC effectiveness. In Model 4, δ_1_ represents the direct effect of digital transformation on total factor productivity, and δ_2_ is the effect of IC on total factor productivity. If δ_1_ is less than α_1_, it indicates that effective IC has a mediating effect for the impact of digital transformation on total factor productivity, meaning that digital transformation enhances IC effectiveness, then increases total factor productivity. In line with Li (2022) [38], Li and Zhao (2022) [39], briefly, Fig 1 describes the mediating effect process.

## 4. Descriptive statistic and correlation

### 4.1 Descriptive statistic

Table 2 presents the descriptive statistics. For the explained variable, the maximum (minimum) of TFP_Fe is 15.727 (7.826), and the mean (standard deviation) is 11.593 (1.335). Among enterprises, there is a certain differences in total factor productivity. For the explanatory variable, the mean (median) of DIGIT is 0.105 (0.065), and the maximum (minimum) is 0.449 (0.010). More than half of enterprises’ digital transformation is lower than the average, and it is difficult to achieve digital scale effect. Digital transformation process varies greatly, and some enterprises’ digital transformation needs to be further promoted. For the mediating variable, the mean (median) of IC is 0.645 (0.666), and the maximum (minimum) is 0.823 (0.000). On the whole, IC effectiveness is good. However, there are great differences among enterprises, and a few enterprises’ IC is in an invalid state.

**Table 2:** Descriptive statistics.

Variable	Mean	Median	Maximum	Minimum	Deviation	Observations
TFP_Fe	11.593	11.437	15.727	7.826	1.335	17574
DIGIT	0.105	0.065	0.449	0.010	0.102	17574
IC	0.645	0.666	0.823	0.000	0.115	17574
LEV	41.011	40.303	86.927	5.676	19.339	17574
TURNOVER	0.656	0.568	2.467	0.119	0.399	17574
ROA	4.396	4.110	21.359	-20.569	5.947	17574
TQ	2.076	1.670	7.969	0.862	1.255	17574
INDED	0.375	0.333	0.571	0.333	0.053	17574
R&D	4.811	3.710	26.520	0.030	4.654	17574
Concen	7.532	3.444	52.930	1.024	10.457	17574
Age	18.937	18.543	35.707	8.041	5.491	17574
LnSALARY	5.288	5.257	7.252	3.708	0.689	17574
LnASSET	22.275	22.073	26.186	20.083	1.275	17574
Big4	0.052	0.000	1.000	0.000	0.223	17574
SOE	0.311	0.000	1.000	0.000	0.463	17574

For the control variables, the maxima (minima) of LEV, TURNOVER, ROA, TQ, R&D, LnSALARY, and LnASSET are 86.93% (5.68%), 2.467 (0.119), 21.36% (-20.57%), 7.969 (0.862), 26.52% (0.03%), 7.252 (3.708), and 26.186 (20.083). Among enterprises, significant differences exist in asset size, debt pressure, asset turnover and profitability, growth, R&D investment, and executive compensation. The minimum of INDED is 0.333, in line with the regulation that “at least one third of the board members should be independent directors in a listed company,” issued by China Securities Regulatory Commission. And the mean of Concen is 7.532, indicating that “one dominant share” exists objectively. Besides, corporate average age is 18.937. On average, the proportion that annual financial reports are audited by the international “Big four” accounting firms is 5.22%. State-owned enterprises account for 31.10%. In general, the sample is well differentiated, providing a useful basis for regression.

### 4.2 Correlation

Table 3 reports the pairwise correlations. In Models 2 and 4, DIGIT is negatively and significantly correlated with TFP_Fe (-0.072, *p*< 0.01), suggesting that the effect of digital transformation on total factor productivity needs to be further examined by regression analyses, with considering other factors. In Model 3, DIGIT is positively and significantly correlated with IC (0.028, *p*< 0.01), preliminarily supporting Hypothesis 2 above, implying that digital transformation enhances IC effectiveness. With digital process, the open, sharing and flexible business modes reduce irrational decision-making, helping to enhance IC execution. In Model 4, IC is positively and significantly correlated with TFP_Fe (0.146, *p*< 0.01), suggesting that effective IC has a promoting effect on total factor productivity.

**Table 3:** Pairwise correlations.

Variable	TFP_Fe	DIGIT	IC	LEV	TURNOVER	ROA	TQ	INDED	R&D	Concen	Age	LnSALARY	LnASSET	Big4	SOE
TFP_Fe	1.000														
DIGIT	-0.072***	1.000													
IC	0.146***	0.028***	1.000												
LEV	0.555***	-0.104***	-0.075***	1.000											
TURNOVER	0.477***	-0.024***	0.152***	0.188***	1.000										
ROA	0.067***	0.003	0.353***	-0.351***	0.188***	1.000									
TQ	-0.317***	0.139***	-0.004	-0.313***	-0.037***	0.237***	1.000								
INDED	-0.005	0.058***	0.021***	-0.010	-0.031***	-0.002	0.035***	1.000							
R&D	-0.368***	0.444***	-0.015**	-0.319***	-0.297***	-0.003	0.276***	0.072***	1.000						
Concen	0.121***	-0.113***	-0.016**	0.123***	0.074***	-0.052***	-0.061***	0.007	-0.143***	1.000					
Age	0.200***	0.056***	-0.061***	0.150***	0.038***	-0.056***	-0.057***	-0.052***	-0.085***	-0.008	1.000				
LnSALARY	0.459***	0.175***	0.105***	0.141***	0.139***	0.180***	-0.022***	0.023***	0.076***	-0.105***	0.230***	1.000			
LnASSET	0.908***	-0.076***	0.100***	0.547***	0.112***	-0.014*	-0.346***	0.011	-0.267***	0.102***	0.207***	0.452***	1.000		
Big4	0.324***	-0.037***	0.086***	0.131***	0.062***	0.033***	-0.076***	0.052***	-0.060***	-0.010	0.004	0.250***	0.343***	1.000	
SOE	0.369***	-0.138***	-0.019**	0.314***	0.085***	-0.127***	-0.151***	-0.046***	-0.221***	0.283***	0.143***	0.019**	0.386***	0.133***	1.000

Note: *** Significant at 1%; ** Significant at 5%; * Significant at 10%.

For the control variables, in Models 2 and 4, LEV (0.555), TURNOVER (0.477), ROA (0.067), Concen (0.121), Age (0.200), LnSALARY (0.459), LnASSET (0.908), Big4 (0.324), SOE (0.369) are positively and significantly correlated with TFP_Fe (*p*< 0.01); and TQ (-0.317), R&D (-0.368) are negatively and significantly correlated with TFP_Fe (*p*< 0.01). In Models 3, TURNOVER (0.152), ROA (0.353), INDED (0.021), LnSALARY (0.105), LnASSET (0.100), Big4 (0.086) are positively and significantly correlated with IC (*p*< 0.01); and LEV (-0.075), Age (-0.061) are negatively and significantly correlated with IC (*p*< 0.01); as are R&D, Concen, and SOE (-0.015, *p*< 0.05; -0.016, *p*< 0.05; -0.019, *p*< 0.05). The correlations ensure the rationality of Models 2 to 4 above. Besides, the maximal correlation is 0.547, between LEV and LnASSET, less than the threshold of 0.800, suggesting that there exists no serious multicollinearity, providing a reliable guarantee for regression.

## 5. Model regression analysis

Without controlling for other factors affecting the explained variables, the descriptive statistics and pairwise correlations are preliminary results, requiring regression analyses to examine the causal relationship between the explanatory variable and explained variables. Panel data is adopted in this study. Mainly, the regression for Panel data includes the mixed OLS method, fixed-effects and random-effects models. The fixed-effects regression has certain information advantages, alleviating the interference from the unobservable factors that do not change with time. Table 4 reports the results with fixed-effect regression for Model 2. Table 5 presents those for Models 3 and 4.

**Table 4:** Regression results for Model 2.

Variable	Model 2		
	(1)	(2)	(3)
	Coef. (S.E.)	Coef. (S.E.)	Coef. (S.E.)
DIGIT	3.218*** (0.140)	0.307*** (0.075)	0.277*** (0.079)
L.LEV		0.090* (0.050)	0.099** (0.050)
L.TURNOVER		0.713*** (0.031)	0.696*** (0.031)
L.ROA		0.308*** (0.110)	0.282*** (0.108)
L.TQ		0.031*** (0.004)	0.042*** (0.005)
INDED		-0.183* (0.111)	-0.198* (0.108)
L.R&D		-0.820*** (0.253)	-0.805*** (0.259)
Concen		-0.013* (0.007)	-0.013** (0.006)
Age		0.037*** (0.003)	-0.010 (0.020)
L.LnSALARY		0.020* (0.012)	0.016 (0.012)
L.LnASSET		0.622*** (0.018)	0.617*** (0.019)
L.Big4		0.057* (0.030)	0.061** (0.030)
SOE		-0.099*** (0.034)	-0.103*** (0.035)
YEAR			YES
IND			YES
Intercept	11.255*** (0.015)	-3.449*** (0.352)	-2.262*** (0.645)
# of obs.	17574	13448	13448
Within_R^2^	0.110	0.670	0.677
F_Value	529.34***	626.81***	298.27***

Note: *** Significant at 1%; ** Significant at 5%; * Significant at 10%.

Robust standard errors in brackets are clustered at corporate level.

**Table 5:** Regression results for Models 3 and 4.

Variable	Model 3			Model 4		
	(1)	(2)	(3)	(4)	(5)	(6)
	Coef. (S.E.)	Coef. (S.E.)	Coef. (S.E.)	Coef. (S.E.)	Coef. (S.E.)	Coef. (S.E.)
DIGIT	-0.047*** (0.016)	0.063*** (0.019)	0.072*** (0.020)	3.228*** (0.140)	0.270*** (0.072)	0.234*** (0.076)
IC				0.224*** (0.056)	0.591*** (0.040)	0.587*** (0.040)
L.LEV		-0.020 (0.016)	-0.019 (0.016)		0.102** (0.048)	0.110** (0.048)
L.TURNOVER		0.022*** (0.007)	0.020*** (0.007)		0.700*** (0.030)	0.684*** (0.030)
L.ROA		0.190*** (0.033)	0.202*** (0.033)		0.195* (0.106)	0.163 (0.105)
L.TQ		0.002* (0.001)	0.002 (0.001)		0.030*** (0.004)	0.041*** (0.005)
INDED		-0.046 (0.038)	-0.049 (0.038)		-0.156 (0.109)	-0.169 (0.106)
L.R&D		0.038 (0.064)	0.036 (0.064)		-0.842*** (0.249)	-0.826*** (0.255)
Concen		-0.004** (0.002)	-0.004** (0.002)		-0.010 (0.006)	-0.011* (0.006)
Age		0.001 (0.001)	0.001 (0.005)		0.037*** (0.003)	-0.011 (0.019)
L.LnSALARY		0.004 (0.004)	0.003 (0.004)		0.018 (0.011)	0.014 (0.011)
L.LnASSET		-0.017*** (0.005)	-0.018*** (0.005)		0.632*** (0.017)	0.627*** (0.018)
L.Big4		0.031** (0.013)	0.031** (0.013)		0.039 (0.029)	0.043 (0.029)
SOE		-0.031*** (0.010)	-0.034*** (0.010)		-0.080** (0.032)	-0.083** (0.033)
YEAR			YES			YES
IND			YES			YES
Intercept	0.650*** (0.002)	0.992*** (0.089)	1.009*** (0.178)	11.110*** (0.041)	-4.036*** (0.341)	-2.854*** (0.613)
# of obs.	17574	13448	13448	17574	13448	13448
Within_R^2^	0.001	0.023	0.032	0.112	0.684	0.691
F_Value	8.36***	9.65***	6.61***	266.67***	648.47***	317.77***

Note: *** Significant at 1%; ** Significant at 5%; * Significant at 10%.

Robust standard errors in brackets are clustered at corporate level.

### 5.1 Analyses of Model 2’s regression results

#### 5.1.1 Effect of digital transformation on total factor productivity

In Table 4, columns 1 to 3 report the results without control variables, without annual and industry effects, and with control variables, respectively. From columns 1 to 3, the coefficients on DIGIT are positive and significant (3.218, *p*< 0.01; 0.307, *p*< 0.01; 0.277, *p*< 0.01), indicating that digital transformation has a significant incentive effect on total factor productivity. The development of digital economy helps enterprises integrate existing resources [49]. Supported by digital technologies, digital transformation promotes enterprises to innovate external interaction, communication methods and link channels [50], and broaden information disclosure and transmission [51], thereby innovate value creation and business modes [52]. As an important form of deep integration for digital and real economies, digital transformation improves management efficiency, reduces operation costs, optimizes resource allocation, releases digital dividends, shows “digital vitality,” and effectively facilitates total factor productivity. Hypothesis 1 above is verified. Digital economy has become an important driving force for steady growth and high-quality development.

#### 5.1.2 Analyses of control variables’ regression results

For the control variables, in columns 2 and 3, the coefficients on L.LEV are positive and significant (0.090, *p*< 0.10; 0.099, *p*< 0.05); as are those on L.TURNOVER (0.713, *p*< 0.01; 0.696, *p*< 0.01), on L.ROA (0.308, *p*< 0.01; 0.282, *p*< 0.01), and on L.TQ (0.031, *p*< 0.01; 0.042, *p*< 0.01). Good asset turnover and profitability, corporate growth, and creditor governance effect motivate enterprises to reinforce operational efficiency, thus increasing total factor productivity. And those on L.LnASSET are positive and significant (0.622, *p*< 0.01; 0.617, *p*< 0.01); as are those on L.Big4 (0.057, *p*< 0.10; 0.061, *p*< 0.05). Usually, compared with upstream and downstream enterprises, large-scale ones have stronger financial strength, higher bargaining power, better resources and risk-taking capacity; and high-quality external audit has a good effect on corporate governance [53], and transmits value signals to the capital market [54], which are conducive to enhancing total factor productivity. Also, in column 2, those on Age, and L.LnSALARY are positive and significant (0.037, *p*< 0.01; 0.020, *p*< 0.10). Perhaps, during growth or maturity phases, good operation efficiency contributes to total factor productivity. As corporate leaders, senior executives decide strategic orientation and resource allocation. Good compensation incentives promote them to perform duties diligently and prudently, ameliorating total factor productivity.

However, in columns 2 and 3, those on Concen are negative and significant (-0.013, *p*< 0.10; -0.013, *p*< 0.05). Taking advantage of control rights, the largest shareholder encroaches corporate resources through hidden means such as related-party transactions, reducing capital allocation efficiency, damaging minority shareholders’ interests [55], and adversely affecting total factor productivity. As are those on SOE (-0.099, *p*< 0.01; -0.103, *p*< 0.01). State-owned enterprises enjoy the government support, face lower competition pressure, to a certain extent, lack the enthusiasm to increase total factor productivity. And as are those on INDED (-0.183, *p*< 0.10; -0.198, *p*< 0.10), and on L.R&D (-0.820, *p*< 0.01; -0.805, *p*< 0.01). Maybe, R&D subsidies’ effectiveness is weakened in the enterprises engaging in rent-seeking and political connections [56]. And the regulators need to urge independent directors to perform supervisory duties actively, to utilize R&D investment efficiently, and fully increase total factor productivity.

### 5.2 Analyses of Models 3 and 4’s regression results

#### 5.2.1 Effect of digital transformation on IC effectiveness

In Table 5, for Model 3, columns 1 to 3 report the results without control variables, without annual and industry effects, and with control variables, respectively. In column 1, the coefficient on DIGIT is negative and significant (-0.047, *p*< 0.01). Clearly, this is due to the regression bias with omitted variables. From columns 2 to 3, those on DIGIT are positive and significant (0.063, *p*< 0.01; 0.072, *p*< 0.01), implying that digital transformation has a significant promoting effect on IC effectiveness. Hypothesis 2 above is verified. As an internal management system, IC execution is affected by digital transformation. Digital transformation embeds digital technologies into production chains, breaks traditional management methods, reshapes production structures and modes [57], and promotes management paradigm to achieve substantive innovation, to realize intelligent, precise and efficient development [58]. Digital transformation realizes automated process control, characterized by being difficult to tamper with, authority checks and balances, process embedding, and human-machine collaboration, ensuring orderly operation in various functional departments, improving internal governance, and reducing managers’ irrational behaviors. The management information and decision support systems from digital transformation enhance IC effectiveness.

#### 5.2.2 Mediating effect of IC between digital transformation and total factor productivity

In Table 5, for Model 4, columns 4 to 6 report the results without control variables, without annual and industry effects, and with control variables, respectively. From columns 4 to 6, the coefficients on DIGIT are positive and significant (3.228, *p*< 0.01; 0.270, *p*< 0.01; 0.234, *p*< 0.01). Digital transformation has commercial value [59], improving business processes within organizational boundaries, innovating business modes, broadening paths to create value for stakeholders [60], and increasing total factor productivity. And those on IC are positive and significant (0.224, *p*< 0.01; 0.591, *p*< 0.01; 0.587, *p*< 0.01). Effective IC forms a good supervision and incentive mechanism, improving decision-making, optimizing market feedback, and enhancing total factor productivity. Further, considering that those on DIGIT in Models 2 and 3 are significant (*p*< 0.01), this study believes that effective IC has a significant mediating effect for the impact of digital transformation on total factor productivity. Hypothesis 3 is verified. In digital transformation, the traditional IC focusing on institutional improvement is gradually transformed into the IC system matching digital strategies. Digital transformation facilitates IC construction and risk prevention, and promotes enterprises to achieve high-quality development. Effective IC is a meaningful mediating factor that digital transformation increases total factor productivity.

With reference to Li (2022) [38], Li and Zhao (2022) [39], the non-parametric percentile bootstrap (1000) method for deviation correction is adopted. Further, the confidence interval of β_1_×δ_2_ with 95% confidence is estimated to be [0.031, 0.058], where β_1_×δ_2_ is the product of the effect of DIGIT on IC, and that of IC on TFP_Fe. Approximately, the size of this mediating effect is estimated to be 15.26% (β_1_×δ_2_/α_1_ = (0.072×0.587)/0.277). These results support the positive effect of digital transformation on total factor productivity by enhancing IC effectiveness.

#### 5.2.3 Analyses of control variables’ regression results

For Model 3, in columns 2 and 3, the coefficients on L.TURNOVER are positive and significant (0.022, *p*< 0.01; 0.020, *p*< 0.01); as are those on L.ROA (0.190, *p*< 0.01; 0.202, *p*< 0.01), and on L.Big4 are positive and significant (0.031, *p*< 0.05; 0.031, *p*< 0.05). Good asset turnover and profitability motivate enterprises to facilitate IC effectiveness, to achieve better operating efficiency and effect. And high-quality external audit exerts governance effect [53], intensifies IC effectiveness, and protects stakeholders’ legitimate rights and interests. Also, in column 2, that on L.TQ is positive and significant (0.002, *p*< 0.10). Good development motivates the governance and the management to improve policies and procedures, to enhance IC effectiveness. However, those on Concen is negative and significant (-0.004, *p*< 0.05; -0.004, *p*< 0.05). The possible “tunneling effect” from major shareholders is not conducive to IC construction. As are those on L.LnASSET (-0.017, *p*< 0.01; -0.018, *p*< 0.01), and on SOE (-0.031, *p*< 0.01; -0.034, *p*< 0.01). Usually, state-owned enterprises have a larger assets scale. In Table 3, LnASSET is positively and significantly correlated with SOE (0.386, *p*< 0.01). Possibly, in large-scale enterprises, redundant organizational structure and long control chains cause serious agency problems, and have a negative impact on IC effectiveness.

For Model 4, the coefficients on L.LEV, L.TURNOVER, L.ROA, L.TQ, L.R&D, Concen, Age, L.LnASSET, and SOE are statistically significant, and the conclusions on them are consistent with those from Model 2. And the coefficients on the remaining control variables are not statistically significant.

## 6. Robustness test

### 6.1 Replacing core variables

To further verify Hypotheses 1 to 3, for Models 2 and 4, with reference to Levinsohn and Petrin (2003) [61], Lu and Lian (2012) [44], LP method is adopted to measure total factor productivity, which is expressed as TFP_LP. Further, TFP_Fe is replaced with TFP_LP, which is taken as the explained variable. Meanwhile, it is considered that IC effectiveness in many enterprises tends to improve over time [39]. To mitigate the possible adverse interference of time trend on analyses, in line with Li and Zhao (2022) [39], according to industry-annual standard, DIB · IC index is ranked from low to high, and expressed as RIC, to measure IC effectiveness. In Models 3 and 4, RIC is taken as the explained and mediating variables, respectively. The higher the value of RIC, the more effective the IC of the enterprise within same year and industry. Moreover, to mitigate the adverse impacts of dimensional differences, RIC is normalized based on industry-annual standard. In Table 6, columns 1 to 3 show the results after replacing TFP_Fe with TFP_LP, and IC with RIC.

**Table 6:** Results after replacing core variables.

Variable	(1)	(2)	(3)	(4)	(5)	(6)
	Model 2	Model 3	Model 4	Model 2	Model 3	Model 4
	Coef. (S.E.)	Coef. (S.E.)	Coef. (S.E.)	Coef. (S.E.)	Coef. (S.E.)	Coef. (S.E.)
DIGIT	0.271*** (0.077)	0.210*** (0.062)	0.213*** (0.072)			
RIC			0.274*** (0.011)			
L.DDIGIT				0.124** (0.062)	0.037* (0.022)	0.104* (0.061)
MIC						0.538*** (0.042)
L.LEV	0.059 (0.047)	-0.076** (0.036)	0.080* (0.045)	0.031 (0.058)	-0.016 (0.017)	0.040 (0.056)
L.TURNOVER	0.704*** (0.029)	0.062*** (0.019)	0.687*** (0.028)	0.632*** (0.038)	0.026*** (0.009)	0.618*** (0.037)
L.ROA	0.264*** (0.101)	0.083 (0.075)	0.240** (0.096)	0.251** (0.117)	0.111*** (0.034)	0.191* (0.113)
L.TQ	0.036*** (0.004)	0.011*** (0.003)	0.033*** (0.004)	0.036*** (0.005)	0.004*** (0.001)	0.034*** (0.005)
INDED	-0.188* (0.105)	-0.084 (0.093)	-0.165* (0.100)	-0.177 (0.126)	-0.080* (0.047)	-0.134 (0.124)
L.R&D	-0.944*** (0.227)	0.186 (0.142)	-0.994*** (0.219)	-0.834*** (0.266)	-0.007 (0.082)	-0.830*** (0.258)
Concen	-0.010* (0.006)	-0.012*** (0.005)	-0.006 (0.005)	-0.009 (0.006)	-0.003* (0.002)	-0.007 (0.006)
Age	-0.011 (0.016)	-0.004 (0.013)	-0.010 (0.015)	-0.002 (0.015)	-0.001 (0.004)	-0.002 (0.014)
L.LnSALARY	0.019* (0.011)	-0.012 (0.010)	0.022** (0.011)	0.007 (0.013)	-0.001 (0.004)	0.007 (0.013)
L.LnASSET	0.480*** (0.017)	-0.025** (0.011)	0.487*** (0.015)	0.441*** (0.021)	-0.012** (0.006)	0.448*** (0.020)
L.Big4	0.060** (0.029)	0.117*** (0.034)	0.028 (0.028)	0.057 (0.035)	0.035*** (0.013)	0.039 (0.035)
SOE	-0.099*** (0.032)	-0.078*** (0.025)	-0.077*** (0.030)	-0.102*** (0.035)	-0.033*** (0.012)	-0.085*** (0.033)
YEAR	YES	YES	YES	YES	YES	YES
IND	YES	YES	YES	YES	YES	YES
Intercept	-1.677*** (0.536)	1.383*** (0.422)	-2.058*** (0.504)	-0.672 (0.636)	0.258 (0.189)	-0.810 (0.612)
# of obs.	13412	13403	13403	10370	10370	10370
Within_R^2^	0.617	0.014	0.647	0.571	0.019	0.588
F_Value	214.70***	3.28***	243.72***	146.03***	3.34***	159.36***

Note: *** Significant at 1%; ** Significant at 5%; * Significant at 10%.

() denotes robust standard errors clustered at corporate level.

Effective IC restrains executive behaviors, reduces agency costs, prevents, detects and corrects material misstatements in financial statements [62]. IC is a series of management activities, and dynamic process with constant development and change [63]. There may be inherent differences in corporate IC construction in different industries. To eliminate the interference caused by the differences in IC effectiveness among industries, with reference to Li (2022) [38], based on industry-annual standard, IC is de-averaged to obtain the adjusted IC effectiveness (MIC). Meanwhile, with current period relative to previous one, the first-order difference of DIGIT is calculated, and expressed as DDIGIT. Given that the impacts of digital transformation on IC, and total factor productivity may have a certain transmission time, the first-order lag of DDIGIT is adopted for analyses. In Table **6**, columns 4 to 6 show those after replacing TFP_Fe with TFP_LP, and IC with MIC.

From columns 1 to 3, the coefficients on DIGIT are positive and significant (0.271, *p*< 0.01; 0.210, *p*< 0.01; 0.213, *p*< 0.01); as are those on L.DDIGIT (0.124, *p*< 0.05; 0.037, *p*< 0.10; 0.104, *p*< 0.10) from columns 4 to 6. Digital transformation integrates production, and management with digital technologies, integrates internal and external resources, splits and reorganizes digital assets, realizes product and service innovation [64], reshapes organizational structure and development strategies, and enhances total factor productivity. Moreover, digital technologies improve the regulatory mechanism for the capital market, and promote corporate governance [65], reducing the possibility that the management overrides IC execution, to ensure IC effectiveness. Again, Hypotheses 1 and 2 are verified.

In columns 3 and 6, those on RIC, and MIC are positive and significant (0.274, *p*< 0.01; 0.538, *p*< 0.01). Effective IC increases total factor productivity, and promotes corporate high-quality development. Given those on DIGIT, and L.DDIGIT from Models 2 to 4, and those on RIC, and MIC in Model 4, this study argues that, as the focus of digital economy development, digital transformation takes artificial intelligence, big data, cloud computing, and other digital technologies as important starting points, to ameliorate IC effectiveness, achieve dynamic and efficiency reform, and then increase total factor productivity. Therefore, effective IC presents a significant mediating effect in the process that digital transformation increases total factor productivity. For columns 1 to 3, and 4 to 6, Sobel tests show that the mediating effect sizes are 9.52%, and 13.12%, respectively. Again, Hypothesis 3 is verified.

For the control variables, in Models 2 and 4, the conclusions on L.LEV, L.TURNOVER, L.ROA, L.TQ, INDED, L.R&D, Concen, L.LnSALARY, L.LnASSET, L.Big4, and SOE are consistent with those from Tables 4 or 5. In Model 3, the conclusions on L.TURNOVER, L.ROA, L.TQ, Concen, L.LnASSET, L.Big4, and SOE are consistent with those from Table 5. Besides, in column 2, the coefficient on L.LEV is negative and significant (-0.076, *p*< 0.05). Excessive debt pressure is not conducive to IC execution. In column 5, that on INDED is negative and significant (-0.080, *p*< 0.10), suggesting that it is necessary for the regulators to urge independent directors to be diligent, and supervise enterprises to intensify IC execution.

### 6.2 Instrumental variable method

Uncertainties exist in corporate digital transformation. The degree of digital mining needs to be considered urgently [66]. It is necessary to rely on organizational process reform to better carry out digital transformation [26], suggesting that there may be reverse causality between total factor productivity and digital transformation, IC and digital transformation. To mitigate the endogeneity caused by reverse causation, with reference to Liu et al. (2021) [67], Instrumental variable method is adopted for analyses. Meanwhile, based on diversification measure, with reference to Wu et al. (2021) [45], and Li et al. (2023) [47], Python technologies are utilized to summarize the occurrence frequencies of the characteristic words related to artificial intelligence, blockchain, cloud computing, big data and digital technology applications from annual reports. The greater the total frequencies of these characteristic words, the higher the degree of digital transformation. Since the data presents obvious right-skew distribution, this study adds 1 to the total frequencies, and takes the natural logarithm as DCG, to measure the degree of digital transformation again.

Appropriate instrumental variables are highly correlated with the endogenous variable, and not with explained variables. With reference to Xiao et al. (2021) [46], the number of landline telephones per 100 people (Telephone), and that of post offices per 10000 people (Post) in the region where the enterprise is located in 1984 are taken as the instrumental variables. The historical communication modes have an important impact on corporate digital transformation, from digital base and social identity. And Telephone and Post do not directly affect total factor productivity and IC effectiveness. Thus, as instrumental variables, Telephone and Post meet correlation and exogeneity. While Panel data is adopted in this study, Telephone and Post belong to cross-sectional data, and which cannot be matched to each other. With reference to Nunn and Qian (2014) [68], the first-order lag of the number of national Internet users (Internet) is introduced to construct Panel instrumental variables. Specifically, Internet and Telephone, Internet and Post are adopted to construct interaction terms, respectively, and expressed as IV_Telephone and IV_Post. Further, Two Stage Least Square (2SLS) regression is conducted, to examine in depth the impacts of digital transformation on IC effectiveness, and total factor productivity.

Table 7 reports the 2SLS results for Models 2 to 4. At the bottom, the coefficients on IV_Telephone, and IV_Post are the first-stage results. From columns 1 to 3, those on IV_Telephone are positive and significant (0.012, *p*< 0.01); as are those on IV_Post (0.018, *p*< 0.01). The historical communication has a significant impact on corporate digital transformation. In accordance with Kleibergen-Paap rk LM statistic (99.627, *p* = 0.000; 99.627, *p* = 0.000; 98.190, *p* = 0.000), Kleibergen-Paap rk Wald F statistic (46.968> 19.930; 46.968> 19.930; 46.263> 19.930), implying that there are no underidentification, and weak identification; and Hansen J statistic (0.075, *p*> 0.10; 1.668, *p*> 0.10; 0.003, *p*> 0.10), indicating that the hypothesis that both instrumental variables are exogenous cannot be rejected at 10% significance. Therefrom, the instrumental variables are valid.

**Table 7:** Test results for instrumental variable method.

Variable	(1)	(2)	(3)
	Model 2	Model 3	Model 4
	Coef. (S.E.)	Coef. (S.E.)	Coef. (S.E.)
DCG	0.087*** (0.028)	0.018** (0.008)	0.075*** (0.028)
IC			0.631*** (0.041)
L.LEV	0.151*** (0.028)	-0.041*** (0.008)	0.177*** (0.027)
L.TURNOVER	1.125*** (0.017)	0.029*** (0.004)	1.107*** (0.017)
L.ROA	0.509*** (0.089)	0.328*** (0.029)	0.301*** (0.088)
L.TQ	0.017*** (0.004)	-0.002* (0.001)	0.018*** (0.004)
INDED	-0.233*** (0.066)	0.015 (0.019)	-0.243*** (0.064)
L.R&D	-1.695*** (0.220)	-0.004 (0.061)	-1.693*** (0.215)
Concen	-0.001 (0.003)	0.001 (0.001)	-0.001 (0.003)
Age	-0.024*** (0.007)	-0.005*** (0.002)	-0.021*** (0.007)
L.LnSALARY	0.027*** (0.007)	-0.001 (0.002)	0.027*** (0.007)
L.LnASSET	0.865*** (0.005)	0.008*** (0.001)	0.860*** (0.005)
L.Big4	0.048*** (0.016)	0.030*** (0.005)	0.029* (0.015)
SOE	0.026** (0.013)	0.008** (0.004)	0.021 (0.013)
YEAR	YES	YES	YES
IND	YES	YES	YES
Intercept	-8.731*** (0.114)	0.442*** (0.031)	-9.010*** (0.115)
# of obs.	11391	11391	11391
F_Value	3852.83***	21.32***	3941.11***
IV_Telephone	0.012*** (0.001)	0.012*** (0.001)	0.012*** (0.001)
IV_Post	0.018*** (0.005)	0.018*** (0.005)	0.018*** (0.005)
Kleibergen-Paap rk LM statistic	99.627 [0.000]	99.627 [0.000]	98.190 [0.000]
Kleibergen-Paap rk Wald F statistic	46.968 {19.930}	46.968 {19.930}	46.263 {19.930}
Hansen J statistic	0.075 [0.784]	1.668 [0.197]	0.003 [0.954]

Note: *** Significant at 1%; ** Significant at 5%; * Significant at 10%.

() denotes robust standard errors clustered at corporate level.

{} denotes Stock-Yogo weak ID test critical value at 10%.

[] denotes *p*-values.

In the second-stage regression, from Models 2 to 4, the coefficients on DCG are positive and significant (0.087, *p*< 0.01; 0.018, *p*< 0.05; 0.075, *p*< 0.01). Digital construction promotes corporate fundamentals’ transformation and reshaping, seeking new growth points in the changing business environment, reducing handling errors, improving total factor productivity, and IC effectiveness. In Model 4, that on IC is positive and significant (0.631, *p*< 0.01). Effective IC increases total factor productivity. From those on DCG in Models 2 to 4, and that on IC in Model 4, this study holds that digital transformation becomes a momentous force to ameliorate IC effectiveness, achieve dynamic and efficiency changes, then increase total factor productivity.

For the control variables, in Models 2 and 4, the conclusions on L.LEV, L.TURNOVER, L.ROA, L.TQ, INDED, L.R&D, L.LnSALARY, L.LnASSET, and L.Big4 are consistent with those from Tables 4 or 5. However, the coefficients on Age are negative and significant (-0.024, *p*< 0.01; -0.021, *p*< 0.01), different from those from Tables 4 and 5. From life cycle perspective, when a company enters recession, it suffers from lacking operational vitalities, impeding total factor productivity. Besides, in Model 2, that on SOE are positive and significant (0.026, *p*< 0.05), different from that from Table 4. Perhaps, based on policy and resource advantages, state-owned enterprises have abundant talent reserves and operational resources, to increase total factor productivity.

In Model 3, the conclusions on L.LEV, L.TURNOVER, L.ROA, and L.Big4 are consistent with those from Tables 5 or 6. However, the coefficient on L.TQ is negative and significant (-0.002, *p*< 0.10), and those on L.LnASSET, and SOE are positive and significant (0.008, *p*< 0.01; 0.008, *p*< 0.05), different from those from Table 5. Maybe, excessive growth intensifies operational risks, which are not conducive to strengthening IC construction. Compared with non-state-owned enterprises, larger-scale state-owned ones shoulder multiple goals, such as creating profits, strategic support, and fulfilling social responsibilities, and more incline to reinforce IC construction. Thereby, risks may be weakened in response to economic policy uncertainty [69]. Besides, that on Age is negative and significant (-0.005, *p*< 0.01). In recession stage, to a certain extent, enterprises lack the enthusiasm to ameliorate IC execution.

### 6.3 Heckman two-step model

With vigorous digital economy, more traditional enterprises are faced with the important challenge of reconstructing operation mode, and development strategy [70]. From practice perspective, digital transformation process is gradual [12, 13]. With steady digital transformation, the high costs and continuous resource input for digital construction break through stable organization operation, leading to a “preference reversal” in enterprises’ attitudes towards transformation behaviors, and shifting from risk preference to risk avoidance [71]. Therefore, digital transformation has a certain degree of “self-selection”problem. To mitigate the endogeneity caused by this “self-selection”as much as possible, Heckman two-step model is adopted to test again.

In the first step, the Probit Model 5 is constructed, to estimate the inverse Mills ratio (InvMills). With reference to Li (2022) [38], Li and Zhao (2022) [39], a dummy variable (DDIG) is set as the explained variable. Based on industry-annual standard, the mean of DIGIT is estimated, and expressed as MDIG. If DIGIT is higher than MDIG, DDIG is equal to 1, implying that the degree of digital transformation is higher. Otherwise, DDIG equals to 0, meaning that the degree of digital transformation is lower. Meanwhile, in Model 5, IV_Telephone and IV_Post are introduced as the instrumental variables, to meet the exclusion restriction. IV_Telephone and IV_Post have the same meanings as in Section 6.2. In the second step, the InvMills estimated in first step is introduced into Models 2 to 4 as a control variable, respectively. Moreover, based on diversification evaluation, TFP_LP is used as the explained variable in Models 2 and 4. On this basis, the impacts of digital transformation on total factor productivity, and IC effectiveness are examined again.

Model 5.


$$\begin{array}{l} DDIG_{i,t}=\lambda_{0}+\lambda_{1}LEV_{i,t-1}+\lambda_{2}TURNOVER_{i,t-1}+\lambda_{3}ROA_{i,t-1}+\lambda_{4}TQ_{i,t-1}+\lambda_{5}INDED_{i,t}+\\ \;\lambda_{6}R\&D_{i,t-1}+\lambda_{7}Concen_{i,t}+\lambda_{8}Age_{i,t}+\lambda_{9}LnSALARY_{i,t-1}+\lambda_{10}LnASSET_{i,t-1}+\\ \;\lambda_{11}Big4_{i,t-1}+\lambda_{12}SOE_{i,t}+\lambda_{13}IV\_Telephone_{i,t}+\lambda_{14}IV\_Post_{i,t}+\\ \;\lambda_{15}\sum_{t}YEAR+\lambda_{16}\sum_{t}IND+\varepsilon_{i,t} \end{array}$$ (6)

In Table 8, in the first-step regression, the coefficients on IV_Telephone are statistically significant (0.014, *p*< 0.01); as are those on IV_Post (0.012, *p*< 0.01), implying that the instrumental variables introduced for sample selection regression are valid. For the second-step regression, in columns 1 and 3, those on InvMills are statistically significant (-0.226, *p*< 0.01; -0.235, *p*< 0.01), meaning that the “self-selection” exists in the sample, to a certain extent. And the regression bias caused by the “self-selection” can be alleviated by Heckman two-step model.

**Table 8:** Test results for Heckman two-step model.

Variable	(1)	(2)	(3)
	Model 2	Model 3	Model 4
	Coef. (S.E.)	Coef. (S.E.)	Coef. (S.E.)
DIGIT	0.388*** (0.074)	0.033* (0.018)	0.363*** (0.073)
IC			0.759*** (0.061)
L.LEV	0.114** (0.046)	-0.038*** (0.011)	0.143*** (0.046)
L.TURNOVER	1.111*** (0.023)	0.043*** (0.005)	1.078*** (0.023)
L.ROA	0.226* (0.123)	0.235*** (0.029)	0.048 (0.123)
L.TQ	0.030*** (0.006)	-0.001 (0.001)	0.030*** (0.006)
INDED	-0.119 (0.108)	0.031 (0.025)	-0.143 (0.107)
L.R&D	-1.568*** (0.325)	0.119 (0.076)	-1.659*** (0.321)
Concen	-0.009 (0.007)	0.001 (0.002)	-0.009 (0.007)
Age	-0.003** (0.001)	-0.001*** (0.000)	-0.002* (0.001)
L.LnSALARY	-0.003 (0.013)	-0.001 (0.003)	-0.003 (0.013)
L.LnASSET	0.672*** (0.008)	0.010*** (0.002)	0.665*** (0.008)
L.Big4	0.065* (0.034)	0.028*** (0.008)	0.044 (0.033)
SOE	0.051** (0.020)	0.008 (0.005)	0.045** (0.020)
InvMills	-0.226*** (0.066)	0.012 (0.016)	-0.235*** (0.066)
YEAR	YES	YES	YES
IND	YES	YES	YES
Intercept	-6.629*** (0.199)	0.404*** (0.047)	-6.935*** (0.198)
# of obs.	11422	11422	11422
IV_Telephone	0.014*** (0.002)	0.014*** (0.002)	0.014*** (0.002)
IV_Post	0.012*** (0.004)	0.012*** (0.004)	0.012*** (0.004)
Wald_chi^2^	34453.24***	475.15***	35407.73***

Note: *** Significant at 1%; ** Significant at 5%; * Significant at 10%.

() denotes standard errors.

In the second-step regression, from Models 2 to 4, the coefficients on DIGIT are positive and significant (0.388, *p*< 0.01; 0.033, *p*< 0.10; 0.363, *p*< 0.01). In Model 4, that on IC is positive and significant (0.759, *p*< 0.01). The higher the degree of digital transformation, the better the total factor productivity and IC effectiveness. As a development mode embedded digital technologies, digital transformation helps to collect and interpret massive data, reduce internal and external information asymmetry, facilitate IC effectiveness, optimize decision-making and management efficiency, thus enhance total factor productivity, forming the transmission path that digital transformation enhances IC effectiveness, and increases total factor productivity. The integration of digital technologies and the real economy is a prominent driving force for high-quality development.

For the control variables, in Models 2 and 4, the conclusions on L.LEV, L.TURNOVER, L.ROA, L.TQ, L.R&D, Age, L.LnASSET, L.Big4, and SOE are consistent with those from Tables 4, 5 or 7. And in Model 3, the conclusions on L.LEV, L.TURNOVER, L.ROA, Age, L.LnASSET, and L.Big4 are consistent with those from Tables 5, 6 or 7.

### 6.4 Multi-period difference-in-differences (DID) test

Digital economy is an important social phenomenon, as well as an institutional phenomenon, and its foothold lies in corporate digital technological transformation [6]. As a basic system, “policy pilot” is particularly critical to corporate digital transformation. In 2013, the “Broadband China” strategy was put forward, so as to select a total of 120 cities in 2014, 2015, and 2016, to promote broadband construction and other network infrastructure gradually. In line with Zhao et al. (2020) [72], the “Broadband China” strategy is used as an exogenous impact to construct the following multi-period DID Models 6 to 8. From Models 6 to 8, Treat distinguishes the treatment group from control group. If a city where the enterprise is located is included in the “Broadband China” pilot list, it is classified in the treatment group (Treat = 1); otherwise, it is classified in the control group (Treat = 0). Post distinguishes the time when the city is listed in the pilot list, and in current and subsequent years, Post is 1; otherwise, Post is 0. Due to the inconsistent implementation time of the “Broadband China” policy in pilot cities, Treat and Post are not controlled in Models 6 to 8, separately. Table 9 reports the results for the multi-period DID tests.

**Model** 6.


$$\begin{array}{l} TFP\_Fe_{i,t}=\theta_{0}+\theta_{1}Treat_{i,t}\times Post_{i,t}+\theta_{2}LEV_{i,t-1}+\theta_{3}TURNOVER_{i,t-1}+\theta_{4}ROA_{i,t-1}+\theta_{5}TQ_{i,t-1}+\\ \;\theta_{6}INDED_{i,t}+\theta_{7}R\&D_{i,t-1}+\theta_{8}Concen_{i,t}+\theta_{9}Age_{i,t}+\theta_{10}LnSALARY_{i,t-1}+\\ \;\theta_{11}LnASSET_{i,t-1}+\theta_{12}Big4_{i,t-1}+\theta_{13}SOE_{i,t}+\\ \;\theta_{14}\sum_{t}YEAR+\theta_{15}\sum_{t}IND+\varepsilon_{i,t} \end{array}$$ (7)

**Model** 7.


$$\begin{array}{l} IC_{i,t}=\eta_{0}+\eta_{1}Treat_{i,t}\times Post_{i,t}+\eta_{2}LEV_{i,t-1}+\eta_{3}TURNOVER_{i,t-1}+\eta_{4}ROA_{i,t-1}+\eta_{5}TQ_{i,t-1}+\\ \;\eta_{6}INDED_{i,t}+\eta_{7}R\&D_{i,t-1}+\eta_{8}Concen_{i,t}+\eta_{9}Age_{i,t}+\eta_{10}LnSALARY_{i,t-1}+\\ \;\eta_{11}LnASSET_{i,t-1}+\eta_{12}Big4_{i,t-1}+\eta_{13}SOE_{i,t}\\ \;+\eta_{14}\sum_{t}YEAR+\eta_{15}\sum_{t}IND+\varepsilon_{i,t} \end{array}$$ (8)

**Model** 8.


$$\begin{array}{l} TFP\_Fe_{i,t}=\varphi_{0}+\varphi_{1}Treat_{i,t}\times Post_{i,t}+\varphi_{2}IC_{i,t}+\varphi_{3}LEV_{i,t-1}+\varphi_{4}TURNOVER_{i,t-1}+\varphi_{5}ROA_{i,t-1}\\ \;+\varphi_{6}TQ_{i,t-1}+\varphi_{7}INDED_{i,t}+\varphi_{8}R\&D_{i,t-1}+\varphi_{9}Concen_{i,t}+\varphi_{10}Age_{i,t}+\\ \;\varphi_{11}LnSALARY_{i,t-1}+\varphi_{12}LnASSET_{i,t-1}+\varphi_{13}Big4_{i,t-1}+\varphi_{14}SOE_{i,t}+\\ \;\varphi_{15}\sum_{t}YEAR+\varphi_{16}\sum_{t}IND+\varepsilon_{i,t} \end{array}$$ (9)

**Table 9:** Results for multi-period DID tests.

Variable	(1)	(2)	(3)
	Model 6	Model 7	Model 8
	Coef. (S.E.)	Coef. (S.E.)	Coef. (S.E.)
Treat×Post	0.018** (0.007)	0.005** (0.002)	0.015** (0.007)
IC			0.634*** (0.037)
L.LEV	0.131*** (0.025)	-0.036*** (0.008)	0.154*** (0.025)
L.TURNOVER	1.249*** (0.014)	0.036*** (0.003)	1.226*** (0.014)
L.ROA	0.349*** (0.085)	0.356*** (0.029)	0.123 (0.084)
L.TQ	0.021*** (0.004)	-0.002 (0.001)	0.022*** (0.004)
INDED	-0.139*** (0.053)	0.027 (0.017)	-0.156*** (0.052)
L.R&D	-0.949*** (0.102)	0.137*** (0.027)	-1.036*** (0.100)
Concen	-0.004 (0.003)	-0.001 (0.001)	-0.003 (0.003)
Age	-0.003*** (0.001)	-0.001*** (0.000)	-0.003*** (0.001)
L.LnSALARY	0.032*** (0.006)	0.001 (0.002)	0.031*** (0.006)
L.LnASSET	0.875*** (0.004)	0.009*** (0.001)	0.869*** (0.004)
L.Big4	0.022 (0.013)	0.026*** (0.005)	0.005 (0.013)
SOE	-0.003 (0.008)	0.004 (0.002)	-0.005 (0.007)
YEAR	YES	YES	YES
IND	YES	YES	YES
Intercept	-8.913*** (0.107)	0.436*** (0.027)	-9.189*** (0.108)
# of obs.	11837	11837	11837
Adj_R^2^	0.940	0.085	0.943
F_Value	4445.45***	21.39***	4450.50***

Note: *** Significant at 1%; ** Significant at 5%; * Significant at 10%.

Standard errors in brackets are clustered at corporate level.

From Models 6 to 8, based on counterfactual framework, the impacts of digitalization on total factor productivity and IC effectiveness are examined more exogenously. If enterprises are located in pilot cities, digital transformation is faster. In contrast, digital transformation is slower. From columns 1 to 3, the coefficients on Treat×Post are positive and significant (0.018, *p*< 0.05; 0.005, *p*< 0.05; 0.015, *p*< 0.05). Digital transformation reshapes corporate fundamentals, prompts enterprises to find new living space in the fierce market competition, reduces information distortion, and enables total factor productivity and IC effectiveness. In column 3, that on IC is positive and significant (0.634, *p*< 0.01). Effective IC increases total factor productivity, to promote development strategy. Considering those on Treat×Post from Models 6 to 8, and that on IC in Model 8, this study holds that digital transformation facilitates IC construction, realize dynamic change and efficiency change, and increase total factor productivity. Effective IC has a significant mediating effect in the process that digital transformation boosts total factor productivity.

For the control variables, in Models 6 and 8, the conclusions on L.LEV, L.TURNOVER, L.ROA, L.TQ, INDED, L.R&D, Age, L.LnSALARY, and L.LnASSET are consistent with those from Tables 4 or 7. In Model 7, the conclusions on L.LEV, L.TURNOVER, L.ROA, L.R&D, Age, L.LnASSET, and L.Big4 are consistent with those from Tables 5 or 7.

Given that the prerequisite for applying DID method is that the treat and control groups have a parallel trend before policy impact, with reference to La Ferrara et al. (2012) [73], a series of interaction items for periods and Treat are introduced, to construct the following Models 9 and 10, and examine whether the treatment and control groups meets Parallel trend hypothesis. If the city belongs to the treatment group, and in a certain year before it is included in the “Broadband China” pilot list, Before* is equal to 1; otherwise, it is 0. If the city belongs to the treatment group, and in a certain year when it is included in the pilot list, Current is 1; otherwise, it is 0. And if the city belongs to the treatment group, and in a certain year after it is included in the pilot list, After* is 1; otherwise, it is 0.

Model 9.


$$\begin{array}{l} IC_{i,t}=\kappa_{0}+\kappa_{1}Before{*}_{i,t}+\kappa_{2}Current_{i,t}+\kappa_{3}After{*}_{i,t}+\kappa_{4}LEV_{i,t-1}+\kappa_{5}TURNOVER_{i,t-1}+\\ \;\kappa_{6}ROA_{i,t-1}+\kappa_{7}TQ_{i,t-1}+\kappa_{8}INDED_{i,t}+\kappa_{9}R\&D_{i,t-1}+\kappa_{10}Concen_{i,t}+\kappa_{11}Age_{i,t}+\\ \;\kappa_{12}LnSALARY_{i,t-1}+\kappa_{13}LnASSET_{i,t-1}+\kappa_{14}Big4_{i,t-1}+\kappa_{15}SOE_{i,t}+\\ \;\kappa_{16}\sum_{t}YEAR+\kappa_{17}\sum_{t}IND+\varepsilon_{i,t} \end{array}$$ (10)

Model 10.


$$\begin{array}{l} TFP\_Fe_{i,t}=\tau_{0}+\tau_{1}Before{*}_{i,t}+\tau_{2}Current_{i,t}+\tau_{3}After{*}_{i,t}+\tau_{4}IC_{i,t}+\tau_{5}LEV_{i,t-1}+\\ \;\tau_{6}TURNOVER_{i,t-1}+\tau_{7}ROA_{i,t-1}+\tau_{8}TQ_{i,t-1}+\tau_{9}INDED_{i,t}+\\ \;\tau_{10}R\&D_{i,t-1}+\tau_{11}Concen_{i,t}+\tau_{12}Age_{i,t}+\tau_{13}LnSALARY_{i,t-1}+\\ \;\tau_{14}LnASSET_{i,t-1}+\tau_{15}Big4_{i,t-1}+\tau_{16}SOE_{i,t}+\\ \;\tau_{17}\sum_{t}YEAR+\tau_{18}\sum_{t}IND+\varepsilon_{i,t} \end{array}$$ (11)

**Fig 2:**
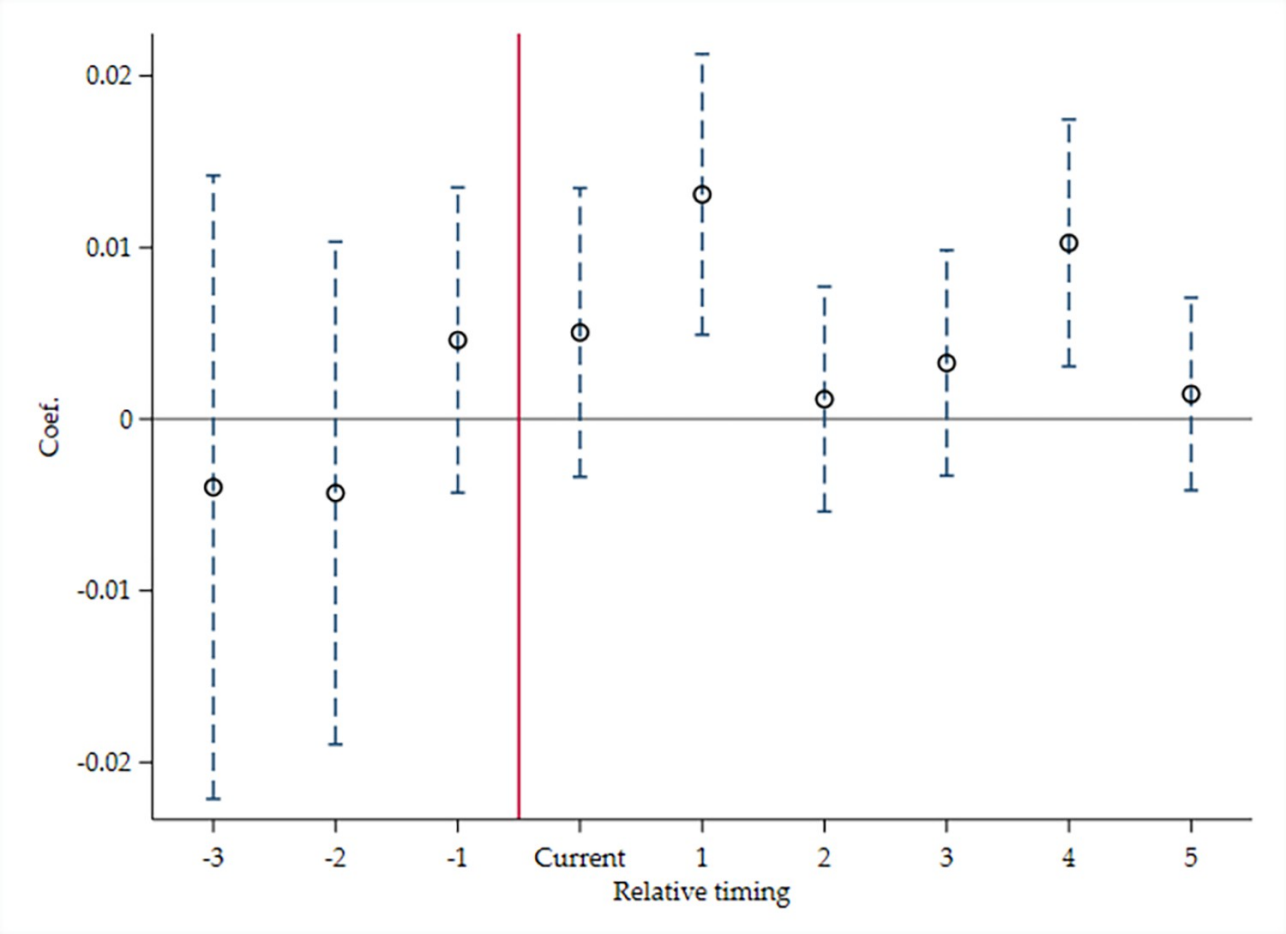
Parallel trend test for Model 9.

**Fig 3:**
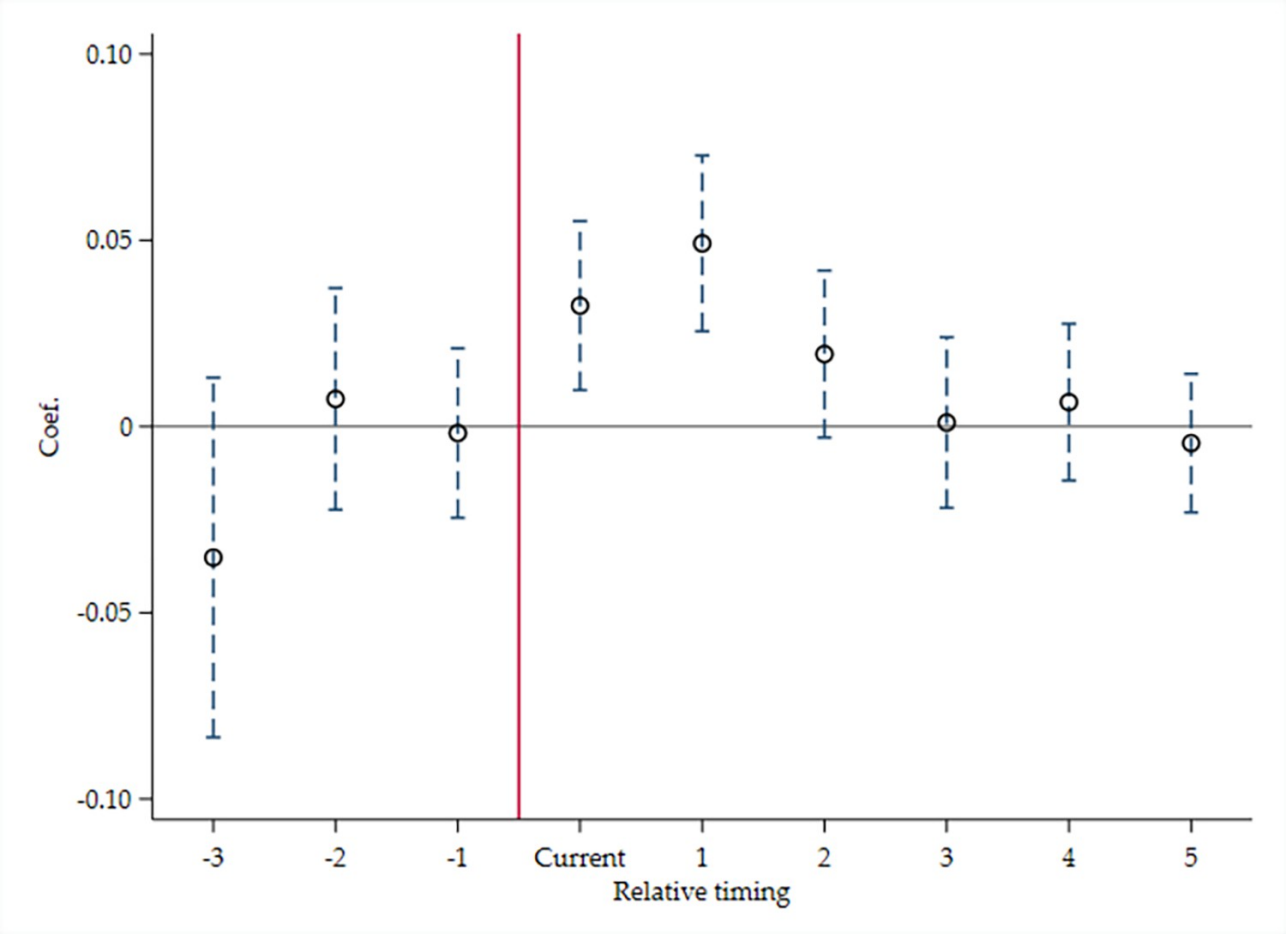
Parallel trend test for Model 10.

For Models 9 and 10, Figs 2 and 3 depict the confidence intervals for the coefficients on Before*, Current, and After* with 90% confidence. On horizontal axis, -3 to 5 indicate the relative time of a observation year relative to the year when the cities are included in the “Broadband China” pilot list. Where, those on Before* are not significant statistically, implying that there are no significant differences between the treatment and control groups’ IC effectiveness, and total factor productivity before the cities are included in the pilot list. In contrast, those on Current, or some After* are significant statistically, meaning distinct differences exist between the two groups, in the year when the cities are included in the pilot list, or after the year. Therefore, the two groups have a parallel trend before the pilot of the “Broadband China” policy, in line with the prerequisite for applying DID method. The results ensure the reliability of the conclusions above. Besides, those Current in Figure, or some After* are not significant statistically. The possible reason is that the “Broadband China”policy has a dynamic effect, with a time lag, and the effect has weakened over time.

## 7. Heterogeneity discussion

### 7.1 Heterogeneity of corporate technological attributes

Digitization is a process in which enterprises apply artificial intelligence, blockchain, cloud computing, big data, internet, and other digital technologies in operation. Digital technologies have a heterogeneous effect on total factor productivity [21]. Enterprises should optimize digital resource allocation in line with own characteristics. High-tech enterprises focus on technological innovation, while non-high-tech ones’ scientific attribute is weaker, and their subjective initiative to conduct digital transformation is lower. In accordance with the “High-tech Industry (Manufacturing) Classification (2017)” and “High-tech Industry (Service Industry) Classification (2018)” issued by Chinese National Bureau of Statistics, those focusing on information transmission, software and information technologies, scientific research and technical services, and manufacturing in pharmaceutical, aviation, spacecraft, computer, communication and other electronic equipments, instrument, and meter are classified as high-tech enterprises (HiTech = 1), and the others are classified as non-high-tech ones (HiTech = 0). With HiTech = 1 and HiTech = 0, grouping tests are performed for Models 2 to 4, to explore the possible heterogeneous effects of digital transformation on total factor productivity, and IC effectiveness. Table 10 shows the results of grouping tests with fixed-effects regression.

**Table 10:** Regression results for HiTech = 1 and HiTech = 0.

Variable	Model 2		Model 3		Model 4	
	(1)	(2)	(3)	(4)	(5)	(6)
	HiTech = 1	HiTech = 0	HiTech = 1	HiTech = 0	HiTech = 1	HiTech = 0
	Coef. (S.E.)	Coef. (S.E.)	Coef. (S.E.)	Coef. (S.E.)	Coef. (S.E.)	Coef. (S.E.)
DIGIT	0.034 (0.080)	0.600*** (0.158)	0.056** (0.027)	0.098*** (0.030)	0.003 (0.077)	0.544*** (0.152)
IC					0.548*** (0.056)	0.566*** (0.053)
L.LEV	0.058 (0.070)	0.174** (0.073)	-0.031 (0.022)	0.011 (0.025)	0.075 (0.068)	0.168** (0.070)
L.TURNOVER	0.711*** (0.056)	0.623*** (0.038)	0.004 (0.011)	0.027*** (0.009)	0.708*** (0.055)	0.608*** (0.037)
L.ROA	0.086 (0.160)	0.433*** (0.150)	0.147*** (0.041)	0.241*** (0.055)	0.006 (0.157)	0.297** (0.144)
L.TQ	0.041*** (0.006)	0.042*** (0.008)	0.002 (0.002)	0.001 (0.002)	0.040*** (0.006)	0.041*** (0.008)
INDED	-0.098 (0.158)	-0.259* (0.146)	-0.053 (0.052)	-0.045 (0.052)	-0.069 (0.154)	-0.234 (0.145)
L.R&D	-1.044*** (0.290)	-0.699* (0.403)	-0.047 (0.080)	0.144 (0.105)	-1.018*** (0.288)	-0.781** (0.398)
Concen	-0.008 (0.008)	-0.017** (0.008)	-0.005 (0.004)	-0.005** (0.002)	-0.006 (0.008)	-0.014* (0.008)
Age	-0.018 (0.025)	-0.0001 (0.033)	-0.001 (0.009)	0.0005 (0.004)	-0.017 (0.021)	-0.001 (0.033)
L.LnSALARY	-0.003 (0.017)	0.019 (0.016)	0.005 (0.006)	-0.001 (0.006)	-0.006 (0.017)	0.020 (0.016)
L.LnASSET	0.620*** (0.024)	0.563*** (0.037)	-0.023*** (0.006)	-0.020** (0.008)	0.632*** (0.023)	0.574*** (0.035)
L.Big4	0.009 (0.035)	0.067 (0.047)	-0.001 (0.016)	0.050*** (0.019)	0.010 (0.035)	0.039 (0.046)
SOE	-0.103*** (0.037)	-0.085 (0.072)	-0.037*** (0.014)	-0.030* (0.017)	-0.082** (0.034)	-0.068 (0.068)
YEAR	YES	YES	YES	YES	YES	YES
IND	YES	YES	YES	YES	YES	YES
Intercept	-1.802** (0.881)	-1.372 (1.085)	1.220*** (0.252)	1.028*** (0.208)	-2.471*** (0.819)	-1.954* (1.057)
# of obs.	6634	6814	6634	6814	6634	6814
Within_R^2^	0.677	0.638	0.037	0.039	0.690	0.652
F_Value	217.31***	286.84***	5.29***	6.52***	222.70***	296.74***

Note: *** Significant at 1%; ** Significant at 5%; * Significant at 10%.

() denotes robust standard errors clustered at corporate level.

For Models 2 and 4, with HiTech = 1, the coefficients on DIGIT are not statistically significant (0.034, *p*> 0.10; 0.003, *p*> 0.10); with HiTech = 0, those are positive and significant (0.600, *p*< 0.01; 0.544, *p*< 0.01). Compared with high-tech enterprises, in non-high-tech ones, digital transformation advances total factor productivity obviously. Digital transformation is a signal that digital technologies empower corporate development, and an indicator of transformation from the traditional industrial automation characterized by “valuing capital over knowledge” to the digital system featuring “valuing knowledge over capital” [74]. High-tech enterprises have intense scientific and technological attributes, and focus on cutting-edge technology development and iterative rejuvenation, while non-high-tech ones may lack sufficient attentions and strategic layout to digital technologies. With HiTech = 1 and HiTech = 0, the means of DIGIT are 0.134, and 0.077; the medians are 0.087, and 0.054, respectively. Both the differences on the means and medians are significant statistically (0.057, *p*< 0.01; 0.033, *p*< 0.01). Therefore, along with extensively applying emerging technologies, non-high-tech ones’ active digital transformation has an obvious marginal promoting effect on total factor productivity.

In Model 3, with HiTech = 1 and HiTech = 0, the coefficients on DIGIT are positive and significant (0.056, *p*< 0.05; 0.098, *p*< 0.01). Fisher’s permutation test shows the difference in those on DIGIT is negative and significant (-0.031, *p*< 0.05). Compared with high-tech enterprises, non-high-tech ones’ digital transformation more significantly ensures IC effectiveness. Digital transformation alleviates traditional operation difficulties [75]. Digital economy promotes the shifts in data analyses from simple sampling to population inspection, and from statements statistics to data mining and intelligent analyses [76]. For non-high-tech ones, digital transformation is reflected as “carbon in snow,” embedding system control function, reducing information system defects, and avoiding that the management overrides IC execution, thus reinforcing IC effectiveness.

Digital transformation motivates enterprises to utilize data resources, increase total factor productivity by enhancing IC effectiveness. With digital development, high-tech enterprises have a stronger technical attribute, and the digital construction efficiency is more obvious. Thereout, the marginal transmission mechanism that digital transformation enhances IC effectiveness, and increases total factor productivity is reflected in non-high-tech ones, but not significantly in high-tech ones. And in non-high-tech ones, effective IC presents a mediating effect size of 9.24% (β_1_×δ_2_/α_1_ = (0.098×0.566)/0.600) for the impact of digital transformation on total factor productivity. Digital technologies have a significant impact on production patterns, requiring enterprises to have keen perception and dynamic capabilities to cope with changes [77]. With technological revolution, for non-high-tech ones, it can be regarded as an effective measure to achieve sustainable development to accelerate digitization, to facilitate IC effectiveness and enhance total factor productivity.

In terms of the control variables, in Models 2 and 4, the conclusions on L.LEV, L.TURNOVER, L.ROA, L.TQ, INDED, L.R&D, Concen, L.LnASSET, and SOE are consistent with those from Tables 4 or 5. In Model 3, the conclusions on L.TURNOVER, L.ROA, Concen, L.LnASSET, L.Big4, and SOE are consistent with those from Table 5 above.

### 7.2 Heterogeneity of executive education backgrounds

Non-high-tech enterprises’ digital transformation has an obvious marginal promoting effect on total factor productivity. In digital era, senior executives need to maintain a highly keen insight and discrimination on digital technologies and new business modes, and timely adjust development strategies [78]. Corporate digital transformation needs support or authorization from senior executives. As corporate leaders, senior executives affect strategic orientation and resource allocation, to a great extent. Executive educational backgrounds’ diversity prompts them to analyze problems rationally from different perspectives [23]. The executives with higher education background have solid theoretical knowledge, stronger systematic thinking and analytical capabilities, and higher cognition and acceptance for cutting-edge hot topics [79]. And the executives with lower educational background did not receive systematic professional training in early stage, but after a long-term work experience, their practical experience and professional knowledge are increasingly enriched. Then, in non-high-tech enterprises, for differentiated executive education, does digital transformation have a heterogeneous effect on total factor productivity, and IC effectiveness?

Senior executives’ education backgrounds reflect their professional knowledge reserves and cognitive capabilities. The heterogeneity in educational backgrounds reflects the degree of educational diversity. With reference to Shen et al. (2023) [80], Herfindahl index (Hijt) is taken to estimate the heterogeneity of executive educational backgrounds. Eq (12) presents the calculation for H_ijt_. Where, P_ijt_ represents the proportion of j-type members in executives for company ***i*** in year ***t***, involving doctor, master, bachelor, junior college, or below, to total executives. Further, a dummy variable for executive educational backgrounds is set, and expressed as HiEdu. If H_ijt_ is higher than its industry-annual mean, HiEdu is equal to 1, indicating that the heterogeneity of executive education backgrounds is higher. Otherwise, HiEdu is 0, implying that the heterogeneity of executive education backgrounds is lower. On this basis, for non-high-tech enterprises, with HiEdu = 1 and HiEdu = 0, from Models 2 to 4, grouping tests are conducted, to examine the possible heterogeneous effects of digital transformation on total factor productivity, and IC effectiveness. Table 11 shows the results with fixed-effects regression.


$$H_{\mathrm{ijt}}=1‐\sum_{j=1}^{n}P_{\mathrm{ijt}}^{2}$$ (12)

**Table 11:** Regression results for HiEdu = 1 and HiEdu = 0.

Variable	Model 2		Model 3		Model 4	
	(1)	(2)	(3)	(4)	(5)	(6)
	HiEdu = 0	HiEdu = 1	HiEdu = 0	HiEdu = 1	HiEdu = 0	HiEdu = 1
	Coef. (S.E.)	Coef. (S.E.)	Coef. (S.E.)	Coef. (S.E.)	Coef. (S.E.)	Coef. (S.E.)
DIGIT	0.286 (0.178)	0.689*** (0.183)	0.042 (0.060)	0.101*** (0.038)	0.267 (0.175)	0.634*** (0.179)
IC					0.433*** (0.083)	0.545*** (0.074)
L.LEV	0.193 (0.151)	0.131 (0.084)	0.073* (0.041)	-0.024 (0.030)	0.161 (0.150)	0.144* (0.082)
L.TURNOVER	0.465*** (0.077)	0.628*** (0.048)	0.024 (0.019)	0.020* (0.012)	0.455*** (0.076)	0.617*** (0.047)
L.ROA	0.493* (0.258)	0.324* (0.186)	0.321*** (0.106)	0.191*** (0.063)	0.354 (0.260)	0.220 (0.177)
L.TQ	0.027*** (0.010)	0.043*** (0.010)	0.001 (0.004)	0.001 (0.003)	0.026*** (0.009)	0.042*** (0.010)
INDED	-0.075 (0.311)	-0.246 (0.158)	-0.151 (0.099)	0.023 (0.061)	-0.010 (0.310)	-0.258 (0.159)
L.R&D	-1.252** (0.532)	-0.607 (0.540)	0.195 (0.221)	0.106 (0.130)	-1.336** (0.541)	-0.665 (0.538)
Concen	0.007 (0.010)	-0.020** (0.010)	-0.007** (0.003)	-0.001 (0.003)	0.010 (0.010)	-0.020** (0.009)
Age	0.086** (0.043)	-0.012 (0.033)	0.005 (0.020)	-0.002 (0.004)	0.084* (0.047)	-0.011 (0.033)
L.LnSALARY	0.005 (0.032)	0.030 (0.018)	-0.014 (0.012)	0.006 (0.007)	0.011 (0.031)	0.026 (0.017)
L.LnASSET	0.454*** (0.087)	0.598*** (0.032)	-0.041** (0.018)	-0.018** (0.007)	0.472*** (0.086)	0.608*** (0.031)
L.Big4	-0.150 (0.099)	0.154** (0.067)	0.074* (0.043)	0.043** (0.018)	-0.182* (0.103)	0.130** (0.064)
SOE	-0.186 (0.121)	-0.092* (0.050)	-0.049 (0.049)	-0.017 (0.015)	-0.165 (0.115)	-0.083* (0.049)
YEAR	YES	YES	YES	YES	YES	YES
IND	YES	YES	YES	YES	YES	YES
Intercept	-0.893 (2.264)	-2.207** (1.050)	1.380** (0.626)	1.017*** (0.195)	-1.490 (2.309)	-2.761*** (1.021)
# of obs.	2294	4519	2294	4519	2294	4519
Within_R^2^	0.573	0.656	0.070	0.037	0.587	0.669
F_Value	64.52***	189.18***	3.63***	3.80***	66.16***	194.71***

Note: *** Significant at 1%; ** Significant at 5%; * Significant at 10%.

() denotes robust standard errors clustered at corporate level.

From Models 2 to 4, with HiEdu = 0, the coefficients on DIGIT are not statistically significant (0.286, *p*> 0.10; 0.042, *p*> 0.10; 0.267, *p*> 0.10); with HiEdu = 1, those are positive and significant (0.689, *p*< 0.01; 0.101, *p*< 0.01; 0.634, *p*< 0.01). Heterogeneous educational backgrounds provide intangible resources such as diversified cognition and management experience for executives to implement digital strategies. With higher heterogeneity of educational backgrounds, the complementary effect of theory and practice enables executives to make correct judgments and decisions [81]. Therefore, with higher heterogeneity of executive educational backgrounds, digital transformation expedites total factor productivity and IC effectiveness.

In Model 4, with HiEdu = 0 and HiEdu = 1, those on IC are positive and significant (0.433, *p*< 0.01; 0.545, *p*< 0.01). Effective IC ameliorates total factor productivity. Based on the above results, in non-high-tech ones, with higher heterogeneity of executive education backgrounds, digital transformation enhances IC effectiveness, and improves total factor productivity. Approximately, this mediating effect size is 7.99% (β_1_×δ_2_/α_1_ = (0.101×0.545)/0.689). However, the transmission mechanism is not significantly reflected with lower heterogeneity of education backgrounds. With higher heterogeneity of educational backgrounds, executives realize “theory plus practice” compound experience and diversified thinking, understand deeply digital technologies’ connotation and potential, solve the difficulties in digital process, to smoothly facilitate the mechanism that digital transformation enhances IC effectiveness, and improves total factor productivity. The differences in educational backgrounds enable executives to play respective advantages, and help digital transformation to engender positive effects.

For the control variables, in Models 2 and 4, the conclusions on L.LEV, L.TURNOVER, L.ROA, L.TQ, L.R&D, Concen, L.LnASSET, and SOE are consistent with those from Tables 4 or 5. In columns 2 and 6, the conclusion on L.Big4 is consistent with that form Table 4. In column 5, the coefficient on L.Big4 is negative and significant (-0.182, *p*< 0.10), different from that from Table 4. Maybe, with lower heterogeneity of executive education backgrounds, it is difficult to optimize decision-making due to scarce differentiated resources, hindering high-quality external audit from exerting governance effect, and adversely affecting total factor productivity. In Model 3, the conclusions on L.TURNOVER, L.ROA, Concen, L.LnASSET, and L.Big4 are consistent with those from Table 4. However, in column 3, the coefficient on L.LEV is positive and significant (0.073, *p*< 0.10), different from that from Table 6. Good creditor governance effect is conducive to augmenting IC effectiveness.

## 8. Conclusion and recommendation

### 8.1 Conclusion

Based on Resource-based theory and IC theory, utilizing the data of China’s listed companies from 2012 to 2021, this study explores the impacts of digital transformation on total factor productivity and IC effectiveness, and examines the internal mechanism among digital transformation, IC and total factor productivity. The results show that digital transformation significantly promotes total factor productivity and IC effectiveness. Effective IC plays a mediating effect for the impact of digital transformation on total factor productivity. In Robustness test, the conclusions are confirmed by Replacing core variables, Instrumental variable method, Heckman two-step model, and Multi-period DID tests. This study elucidates the mediating effect of IC for the impact of digital transformation on total factor productivity, contributing to clarify the action mechanism that digital transformation enables total factor productivity.

Heterogeneity discussion shows that compared with high-tech enterprises, in non-high-tech ones, digital transformation improves total factor productivity, and more significantly enhances IC effectiveness. The transmission mechanism that digital transformation enhances IC effectiveness, and improves total factor productivity is reflected in non-high-tech ones, but not significantly in high-tech ones. Moreover, for non-high-tech ones, with higher heterogeneity of executive educational backgrounds, digital transformation facilitates total factor productivity and IC effectiveness significantly, showing the transmission effect that digital transformation facilitates IC effectiveness, and increases total factor productivity, but not with lower heterogeneity of executive education backgrounds. Heterogeneity discussion enriches the research on digital enablement, conducing to cognize digital transformation rationally, and providing practical enlightenments for achieving high-quality development.

### 8.2 Recommendation

Digitalization is an inevitable process that countries need to embrace. The regulatory authorities improve the institutional environment, promote digital infrastructure construction, facilitate the internet platforms that provide services for digital transformation, to create a good digital environment, and propel corporate digital progress. The application scenarios of emerging technologies such as artificial intelligence, and big data in the market are vigorously constructed, to reduce digital transformation costs, guide enterprises to fortify digital thinking, and actively implement digital transformation. The government policy-makers improve the support system for digital transformation, build a business environment conducive to digital transformation, motivate the precise integration of digital technologies and corporate operation, and expedite the integrated development of digital technologies and the real economy, so as to ameliorate digital governance system and capability, and enhance IC effectiveness and risk prevention. Thus, corporate production and operation can be fully empowered, and total factor productivity can be increased.

Enterprises grasp the new opportunities of digital economy development, take the initiative to carry out the all-round application of digital technologies such as big data, fortify the integration and application of digital resources. Enterprises excavate digital technologies’ powerful energy, enhance data processing and analysis capabilities, cultivate good digital thinking, effectively extract business value from data, and adjust business strategies and operational modes in accordance with business conditions, to further increase total factor productivity. Meanwhile, enterprises establish a digitalized enabling governance mechanism, increase digital resources’ input in IC construction, through embedded digital technologies, monitoring operation processes in real time, so as to improve internal governance efficiency, and fully enhance IC effectiveness. Further, the governance role of digital economy can be brought into play, to promote the transmission mechanism among digital transformation, effective IC, and total factor productivity.

In the context of informatization, intellectualization and digitalization, in non-high-tech enterprises, digital transformation has a significant marginal promoting effect on total factor productivity, and more significantly facilitates IC effectiveness. Non-high-tech ones grasp the historical opportunity of emerging technologies’ extensive application and iterative renewal, actively develop and apply digital technologies, and motivate digital elements’ governance efficiency. And appropriately, the heterogeneity of executive educational backgrounds is enhanced, to form a structure with both highly educated executives with solid theoretical foundation, and team members with rich first-line work experience, realize the “theory plus practice” compound experience and diversified thinking, actively cope with and adapt to digital process. Executives’ cooperation and decision-making capabilities are cultivated, to give full play to respective advantages, integrate and utilize differentiated resources, optimize strategic decisions, give impetus to digital upgrading collaboratively, then enhance internal governance efficiency and total factor productivity, thereby helping the real economy achieve high-quality development.

### 8.3 Limitation and prospect

Digital transformation has become an important measure to achieve healthy and stable development. Based on data availability, this study only adopts the listed enterprises in China as the sample. Likewise, in Europe, more attention should be focused on total factor productivity, to advance growth potentials [82]. Substantial productivity gains follow from further competition-enhancing measures [83]. For instance, COVID-19 had a negative and significant impact on corporate performance, while digital transformation had a positive and significant effect on performance [84], fostering sustainability transition [85]. And effective IC facilitates corporate long-term development strategy. Therefore, it is reasonable to expect that in European capital markets, as an important force to advance dynamic change and efficiency change, digital transformation reinforces IC effectiveness, and enhances total factor productivity, to realize development quality improvement. In future studies, it is necessary to obtain richer data for empirical tests.

Due to limited space, in Heterogeneity discussion, only based on the heterogeneity of corporate technological attributes, and that of executive education backgrounds in non-high-tech enterprises, this study explores the impacts of digital transformation on total factor productivity and IC effectiveness, and the transmission mechanism among digital transformation, IC and total factor productivity. From information dimension, digital transformation conveys positive signals that enterprises are applying new technologies, attracting stakeholders’ attentions, such as media and analysts, and improving the information environment and market expectations [45]. Good information environment improves transactions’ transparency, and mitigates the information asymmetry between majority and minority shareholders. In the future, the possible mediating effects of media reports and analysts’ attentions for the impact of digital transformation on total factor productivity can be considered, as well as the possible mechanism that digital transformation inhibits majority shareholders’ encroachments, and increases total factor productivity, to provide more empirical evidence for promoting the national economy to achieve high-quality development.

## Supporting information

S1 Dataset The data set used in this article for discussion and analysis. (ZIP)

S1 Appendix Keywords related to digitalization. (DOCX)
