# Supplementary material for: Corporate digital transformation, internal control and total factor productivity
Source: PLoS One. 2024 Mar 20;19(3):e0298633. doi: 10.1371/journal.pone.0298633 (PMC10954155; doi:10.1371/journal.pone.0298633)
Supplement: S1 Appendix — (DOCX) [file pone.0298633.s002.docx]

**Appendix** **1.** **Keywords related to digitalization.**

| 1 | Application data | 26 | Computing technique | 51 | Digital communication | 76 | Fintech |
| --- | --- | --- | --- | --- | --- | --- | --- |
| 2 | Application software | 27 | Connectivity | 52 | Digital control | 77 | Fusion architecture |
| 3 | Artificial intelligence | 28 | Control technique | 53 | Digital creativity | 78 | Gateway |
| 4 | Augmented reality | 29 | Core technology | 54 | Digital currency | 79 | Graph calculation |
| 5 | Automatic control | 30 | Cross-media | 55 | Digital management | 80 | Green computing |
| 6 | Automatic driving | 31 | Cyberport | 56 | Digital finance | 81 | Hardware and software |
| 7 | Automation | 32 | Cyberspace | 57 | Digital intelligence | 82 | Heterogeneous data |
| 8 | Base station | 33 | Data analysis | 58 | Digital marketing | 83 | High and new technology |
| 9 | Big data | 34 | Data center | 59 | Digital network | 84 | Human-computer interaction |
| 10 | Billion concurrency | 35 | Data empowerment | 60 | Digital supply chain | 85 | Image understanding |
| 11 | Biometrics | 36 | Data management | 61 | Digital technique | 86 | Industrial chain |
| 12 | Bit | 37 | Data mining | 62 | Digital television | 87 | Industrial internet |
| 13 | Blockchain | 38 | Data network | 63 | Digital terminal | 88 | Industry 4.0 |
| 14 | Brain-like computing | 39 | Data platform | 64 | Digitization | 89 | Industry-university-research |
| 15 | Broadband | 40 | Data processing | 65 | Distributed computation | 90 | Information |
| 16 | Broadband access | 41 | Data science | 66 | Ecological cooperation | 91 | Information flow |
| 17 | Business intelligence | 42 | Data security | 67 | E-commerce mobile internet | 92 | Information industry |
| 18 | Cloud computing | 43 | Data sharing | 68 | Electronic administration | 93 | Information management |
| 19 | Cloud ecology | 44 | Data visualization | 69 | Electronic business | 94 | Information network |
| 20 | Cloud platform | 45 | Database | 70 | Electronic commerce | 95 | Information physical system |
| 21 | Cloud service | 46 | Deep learning | 71 | Electronic information | 96 | Information security |
| 22 | Cloud storage | 47 | Development tools | 72 | Electronic product | 97 | Information system |
| 23 | Cognitive computing | 48 | Differential privacy technology | 73 | Encoding | 98 | Information technology |
| 24 | Communication network | 49 | Digit | 74 | Energy grid | 99 | Informatization |
| 25 | Computer | 50 | Digital business | 75 | Face recognition | 100 | Integrated circuit |

**Appendix 1. *Cont.***

| 101 | Intellectual technology | 126 | Internet business mode | 151 | Mobile internet | 176 | Relational database |
| --- | --- | --- | --- | --- | --- | --- | --- |
| 102 | Intelligence | 127 | Internet ecology | 152 | Mobile payment | 177 | Robot |
| 103 | Intelligent agriculture | 128 | Internet marketing | 153 | Monitoring network | 178 | Satellite communication |
| 104 | Intelligent algorithm | 129 | Internet medical treatment | 154 | Multimedia | 179 | Semantic search |
| 105 | Intelligent cultural travel | 130 | Internet mobile | 155 | Multi-party secure computing | 180 | Server |
| 106 | Intelligent customer service | 131 | Internet mode | 156 | National defense science and technology | 181 | Service network |
| 107 | Intelligent data analysis | 132 | Internet of Things | 157 | Natural language processing | 182 | Smart financial contract |
| 108 | Intelligent energy | 133 | Internet platform | 158 | Navigation system | 183 | Smart home |
| 109 | Intelligent environmental protection | 134 | Internet protocol | 159 | Network connection | 184 | Smart wear |
| 110 | Intelligent grid | 135 | Internet security | 160 | Network coverage | 185 | Speech recognition |
| 111 | Intelligent healthcare | 136 | Internet solution | 161 | Network equipment | 186 | Technical transformation |
| 112 | Intelligent investment adviser | 137 | Internet strategy | 162 | Network facilities | 187 | Technology development |
| 113 | Intelligent machine | 138 | Internet technology | 163 | Network service | 188 | Terminal product |
| 114 | Intelligent manufacturing | 139 | Invention patent | 164 | Networking | 189 | Text mining |
| 115 | Intelligent marketing | 140 | Investment decision aid system | 165 | New industrialization | 190 | Third-party payment |
| 116 | Intelligent network | 141 | Key technology | 166 | Numerical control | 191 | Transducer |
| 117 | Intelligent robot | 142 | Knowledge management | 167 | Online education | 192 | Unicom |
| 118 | Intelligent supply chain | 143 | Live streaming | 168 | Online finance | 193 | Unmanned retail |
| 119 | Intelligent terminal | 144 | Machine | 169 | Online retailing | 194 | Virtual reality |
| 120 | Intelligent transportation | 145 | Machine learning | 170 | Platform economy | 195 | Virtualization |
| 121 | Intelligentize | 146 | Machine learning internet business mode | 171 | Portal network | 196 | Wireless |
| 122 | Internet + | 147 | Management information system | 172 | Portal website | 197 | Wireless network |
| 123 | Internet action | 148 | Man-machine | 173 | Processor |  |  |
| 124 | Internet application | 149 | Memory computing | 174 | Public data |  |  |
| 125 | Internet business | 150 | Mixed reality | 175 | Quantitative finance |  |  |
